# Furry is required for cell movements during gastrulation and functionally interacts with NDR1

**DOI:** 10.1038/s41598-021-86153-x

**Published:** 2021-03-23

**Authors:** Ailen S. Cervino, Bruno Moretti, Carsten Stuckenholz, Hernán E. Grecco, Lance A. Davidson, M. Cecilia Cirio

**Affiliations:** 1 Facultad de Ciencias Exactas y Naturales, Instituto de Fisiología, Biología Molecular y Neurociencias (IFIBYNE-UBA-CONICET), Universidad de Buenos Aires (UBA), C1428EGA Buenos Aires, Argentina; 2 Facultad de Ciencias Exactas y Naturales. Departamento de Física, Instituto de Física de Buenos Aires (IFIBA-UBA-CONICET), Universidad de Buenos Aires (UBA), C1428EGA Buenos Aires, Argentina; 3 Department of Bioengineering, Swanson School of Engineering, University of Pittsburgh, Pittsburgh, PA 15213 USA; 4 Department of Computational and Systems Biology, University of Pittsburgh, Pittsburgh, PA 15260 USA; 5 Department of Developmental Biology, University of Pittsburgh, Pittsburgh, PA 15260 USA

**Keywords:** Developmental biology, Molecular biology

## Abstract

Gastrulation is a key event in animal embryogenesis during which germ layer precursors are rearranged and the embryonic axes are established. Cell polarization is essential during gastrulation, driving asymmetric cell division, cell movements, and cell shape changes. The *furry* (*fry*) gene encodes an evolutionarily conserved protein with a wide variety of cellular functions, including cell polarization and morphogenesis in invertebrates. However, little is known about its function in vertebrate development. Here, we show that in *Xenopus,* Fry plays a role in morphogenetic processes during gastrulation, in addition to its previously described function in the regulation of dorsal mesoderm gene expression. Using morpholino knock-down, we demonstrate a distinct role for Fry in blastopore closure and dorsal axis elongation. Loss of Fry function drastically affects the movement and morphological polarization of cells during gastrulation and disrupts dorsal mesoderm convergent extension, responsible for head-to-tail elongation. Finally, we evaluate a functional interaction between Fry and NDR1 kinase, providing evidence of an evolutionarily conserved complex required for morphogenesis.

## Introduction

Gastrulation is a crucial time in animal development during which major cell and tissue movements shape the basic body plan^1,2^. The morphogenetic movements of gastrulation rearrange the three germ layers precursors, positioning mesodermal cells between outer ectodermal and inner endodermal cells to shape the head-to-tail body axis. In order to break the initial “egg shape” of the embryo, cells need to polarize in a precise and coordinated manner. Cell polarity controls orientated cell division, cell shape changes, as well as cell movement. Additionally, cell polarity regulates the mechanical behaviors of the tissue, e.g. assembly of the extracellular matrix (ECM) during gastrulation and numerous other morphogenetic events^2–4^. The embryo of the frog *Xenopus laevis* is widely used as a model of cell polarization, migration, and morphogenesis due to its unique experimental advantages. The large size of the embryo and its cells allows extensive manipulation and high resolution live microscopy of explant cultures^3,5^.

At the beginning of *Xenopus* gastrulation, the presumptive anterior mesoderm cells located at the dorsal marginal zone (DMZ) roll inward at the midline of the blastopore lip in a process called involution. Involution follows bottle cell contraction and spreads laterally and ventrally leading to the formation of the blastopore, a ring of involuting cells that encircles the yolky vegetal endoderm cells. As involution proceeds, the blastopore progressively decreases in diameter, defining the posterior of the embryo, and closes at the end of gastrulation^2^. Simultaneously, on the dorsal side of the embryo, axial and paraxial mesoderm tissues undergo convergent extension which elongates the anterior–posterior axis and aids blastopore closure. During convergent extension, mesodermal cells polarize and intercalate with each other along the mediolateral axis, narrowing and extending the dorsal midline^6,7^. Gastrulation movements are orchestrated by a small, heterogeneous group of cells with inductive and morphogenetic properties located in the dorsal lip of the blastopore (DBL) of the amphibian gastrula known as the Spemann-Mangold organizer or dorsal organizer. The process of gastrulation is linked to determination of mesodermal cell fates, such that patterning of tissue fates and patterning of cell behavior are interconnected. In fact, numerous transcription factors controlling axis determination later regulate the morphogenetic behavior of the cells in which they are expressed^8–11^.

The Furry (Fry) gene encodes a large protein (~ 330 kDa) that is evolutionarily conserved from yeast to humans. Fry protein is composed of an N-terminal Furry domain (FD) with HEAT/Armadillo repeats followed by five regions without any recognizable functional domains. Additionally in vertebrates, there are two leucine zipper motifs and a coiled-coil motif at the C-terminus^12^. In invertebrates, and in fission and budding yeasts, the phenotypes associated with loss-of-function mutants of Fry orthologs, including *Drosophila* Fry, *C. elegans* Sax-2, *S. pombe* Mor2p and *S. cerevisiae* Tao3p, implicate this protein in the control of cell division, transcriptional asymmetry, cell polarization, and morphogenesis^13–20^. In mammalian cells, Fry was found in association with microtubules regulating chromosome alignment, bipolar spindle formation in mitosis, and in yes-associated protein (YAP) cytoplasmic retention^21–24^. Many of Fry functions are related to its role as an essential scaffolding factor and activator of NDR1 and NDR2 (nuclear Dbf-2-related) protein kinases. Orthologs of NDR1/2, also known as serine threonine kinase 38 (STK38/38L), were found in several species: Tricornered (trc) in *Drosophila*, Sax-1 in *C.elegans*, Orb6p in *S. pombe* and Cbk1p in *S. cerevisiae*^25^. Genetic and physical interactions between Fry and NDR1 have been observed across a broad group of eukaryotes, where Fry protein modulates NDR1 phosphorylation and kinase activity^14,16,21,24–26^. Neither the function of *Xenopus* ortholog of NDR1 nor its physical and functional interaction with Fry have been investigated.

Fry's role in vertebrate development has only been studied in *Xenopus* where it was described as a maternally expressed gene^27^. In the early gastrula embryo, *fry* transcripts are present in the dorsal and ventral tissues and later in the mesoderm and ectoderm derivatives^27,28^. Fry function has been associated with the regulation of microRNAs regulating the expression of genes in the axial mesoderm (prechordal mesoderm and chordamesoderm) of the early gastrula and the development of the pronephric kidney^27,28^. The study by Goto et al., also showed that Fry has axis-inducing activity resulting in a partial secondary axis when overexpressed in ventral blastomeres^27^.

In this study, we investigate the role of Fry in morphogenetic processes that occur during *Xenopus* gastrulation. We describe its expression during gastrulation and, using morpholino knock-down, show that Fry is required for the normal expression patterns of early organizer genes, blastopore closure, and dorsal axis elongation. At the cellular level, loss of Fry function drastically affects the movement, morphological polarization and mediolateral alignment of mesodermal cells during gastrulation. Consistent with these findings, convergent extension of the dorsal mesoderm is impaired in *fry*-depleted embryos. Finally, we explore the participation of NDR1 in the Fry loss-of-function phenotype. Through rescue experiments, we present evidence of a functional interaction between Fry and NDR1 kinase in *Xenopus*, suggesting an evolutionarily conserved involvement of these proteins in morphogenesis.

## Results

### Dorsal *fry* depletion causes axis elongation defects

We and others have previously determined that *fry* is expressed in the involuting mesoderm of the early gastrula, becoming restricted to dorsal tissues and lateral plate mesoderm of neurula, and remaining in somites, notochord, heart, eye, brain and pronephric kidney through tailbud stages^27,28^. We investigated in more detail its expression pattern before and during gastrulation. We found that *fry* transcripts are present almost exclusively in the animal half of the blastula and its expression domain encompasses the marginal zone in early gastrula (Supplementary Fig. S1a,b). By late gastrula, *fry* transcripts are present in the axial mesoderm and the deep layer of the ectoderm, becoming restricted to the notochord, paraxial and lateral mesoderm in neurula (Supplementary Fig. S1c,d). Since we were not able to detect the endogenous protein with available antibodies, we investigated Fry cellular localization in DMZ explants from *fry-GFP* mRNA injected embryos at a dose that did not cause an axis phenotype. Fry-GFP fusion protein^27^ was mainly present in the cytoplasm and at the plasma membrane of dorsal mesodermal cells during gastrulation (Supplementary Fig. S1e). In a previous report, Fry-GFP was detected in nuclei and cytoplasm of isolated mesodermal *Xenopus* cells^27^. This apparent discrepancy with our results might be related to the cellular context and the identity of the imaged cells. Cytoplasm and cell membrane localization of endogenous Fry protein has been reported in other organisms^14,15,21,24^ and nuclear localization was found in *Drosophila* salivary gland and fat body cells^14^. Together, the evidence argues in favor of a highly mobile protein with distinct spatiotemporal cellular localization dependent on cell type and context.

**Figure 1:**
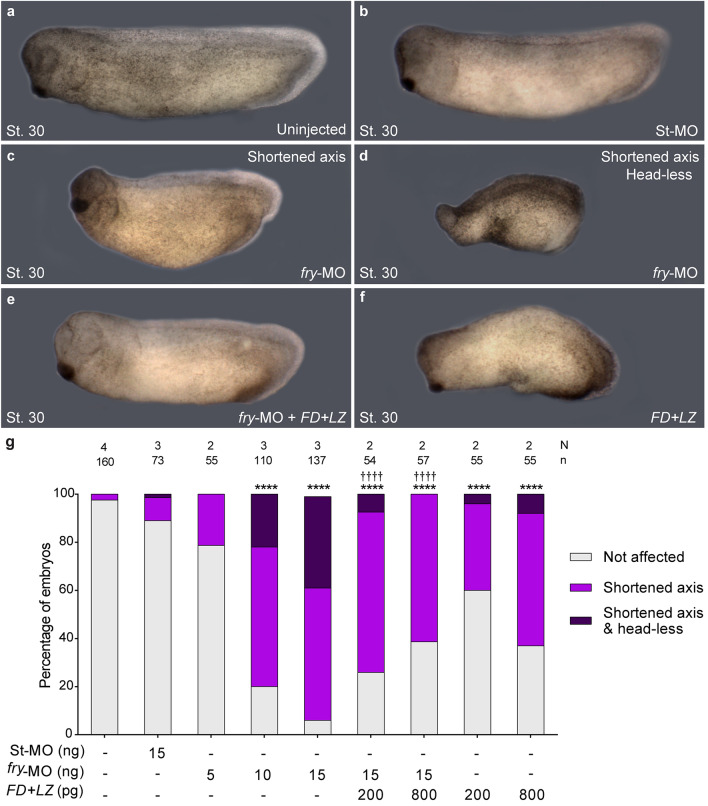
Fry loss-of-function and rescue experiments. 4-cell stage *Xenopus* embryos were injected into both dorsal blastomeres as indicated and fixed at stage 30 (St. 30). (**a**) Uninjected embryo. (**b**) Standard Control morpholino (St-MO) (15 ng) injected embryo. (**c,d**) *fry*-MO (15 ng) injected embryos exhibiting “Shortened axis” or “Shortened axis & Head-less” phenotypes, respectively. (**e**) Rescue experiment; *fry*-MO (15 ng) + *FD* + *LZ* mRNA (800 pg) co-injected embryo. (**f**) *FD* + *LZ* mRNA (800 pg) injected embryo. Representative embryos are shown. (**g**) Quantitation of the percentage of embryos showing the different phenotypes: “Not affected”, “Shortened axis” or “Shortened axis & Head-less”. Embryos were scored as “Shortened axis” when presented < 80% of the body length relative to the average length of control embryos. N: number of independent experiments, n: number of embryos. Data in graph is presented as mean. Statistical significance was evaluated using *Chi*-square test (****,^††††^*p* < 0.0001). * represents the comparison to the uninjected group and † represents the comparison to the *fry*-MO injected group.

We performed loss-of-function experiments by knocking down *fry* translation using a previously-validated antisense morpholino oligonucleotide (*fry-*MO)^27^. As reported, injection of *fry-*MO into both dorsal blastomeres of 4-cell embryos resulted in a shortened anterior–posterior axis and reduced anterior head structures at the tailbud stage (Fig. 1a–d)^27^. This effect was dose dependent and we classified embryos as “Shortened axis” or “Shortened axis & Head-less” as a more severe phenotype when the cement gland and the optic and otic vesicles were absent (Fig. 1c,d,g). Goto et al., have shown that full-length *fry* injection rescues cement gland and axis elongation defects in *fry* morphant embryos^27^. Here, we performed rescue experiments with a short chimeric version of Fry, FD + LZ^27^, that only possesses the N-terminal FD and C-terminal LZ domains and lacks the morpholino target site. In vertebrates, FD and LZ are the only two recognizable functional domains identified in the Fry protein. Together, they have an effect on secondary axis induction very similar to that of full-length *fry*^27^*.* We sought to investigate if these domains were sufficient to rescue the axis elongation phenotype of *fry* morphants. Co-injection of *FD* + *LZ* mRNA led to a significant reduction in the frequency of affected embryos and the severity of the *fry*-depletion phenotype (Fig. 1e,g). These results indicate that the FD and LZ domains can partially compensate for Fry loss-of-function. Intriguingly, dorsal overexpression of *fry* has no effect on axis formation^27^, whereas dorsal overexpression of *FD* + *LZ* mRNA alone causes axis elongation impairment and a head-less phenotype. Taken together, these findings indicate that normal Fry function is required for anterior–posterior axis development (Fig. 1f,g).

### Fry regulates dorsal organizer formation and gastrulation movements

**Figure 2:**
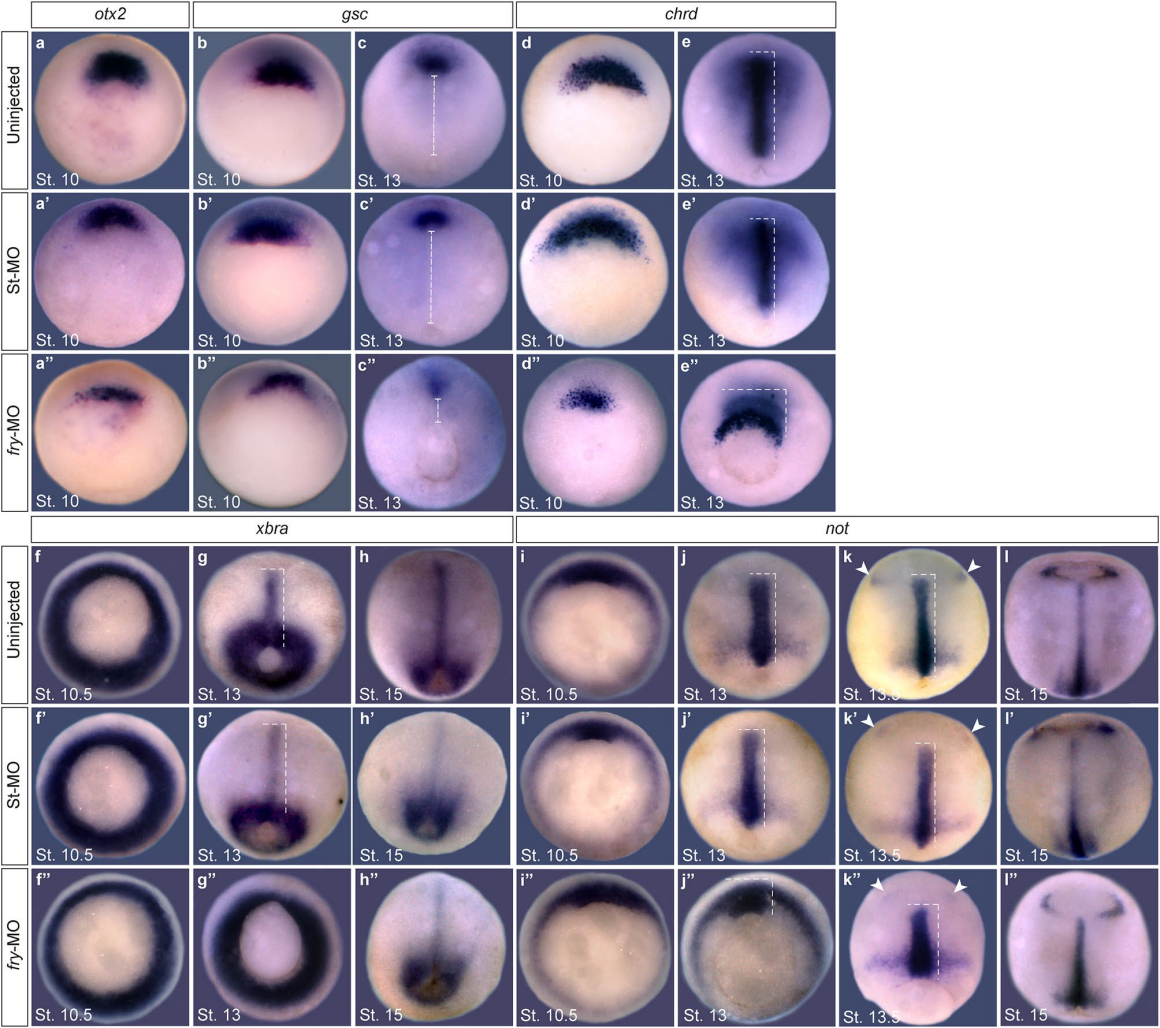
Dorsal *fry*-depletion affects early expression of organizer genes and causes gastrulation defects. (**a**–**l’’**) In situ hybridization of *Xenopus* embryos at the indicated stages. (**a–a’’**) Expression of *otx2* in the presumptive prechordal mesoderm. (**a**) Uninjected embryo (N = 2; n = 33). (**a’**) St-MO injected embryo (N = 2; n = 33; 6% with reduced expression domain) (**a’’**) *fry*-MO injected embryo (N = 2; n = 26; 85% with reduced expression domain). (**b,c’’**) Expression of *gsc* in the presumptive prechordal mesoderm. (**b**) Uninjected St.10 embryo (N = 3; n = 98). (**b’**) *fry*-MO injected St.10 embryo (N = 2; n = 34; 6% with reduced expression domain) (**b’’**) *fry*-MO injected St.10 embryo (N = 3; n = 74; 78% with reduced expression domain). Dorsal is oriented to the top. (**c**) Uninjected St.13 embryo (N = 3; n = 60). (**c’**) St-MO injected St.13 embryo (N = 2; n = 30; 10% with abnormally positioned expression domain). (**c’’**) *fry*-MO injected St.13 embryo (N = 3; n = 62; 87% and 96% with reduced and abnormally positioned expression domain, respectively).The distance between the blastopore and the prechordal mesoderm expressing *gsc* (dashed line) is reduced in the morphants. Dorsal views, anterior is oriented to the top. (**d,e’’**) Expression of *chrd* in the presumptive axial mesoderm. (**d**) Uninjected St. 10 embryo (N = 5; n = 95). (**d’**) St-MO injected St. 10 embryo (N = 2; n = 34; 6% with reduced expression domain) (**d’’**) *fry*-MO injected St. 10 embryo (N = 5; n = 113; 97% with reduced expression domain). Dorsal is oriented to the top. (**e**) Uninjected St.13 embryo (N = 4; n = 128). (**e’**) St-MO injected St.13 embryo (N = 2; n = 30, 10% with abnormally positioned expression domain) (**e’’**) *fry*-MO injected St.13 embryo (N = 4; n = 115, 92% and 95% with reduced and abnormally positioned expression domain, respectively). Dorsal views, anterior is oriented to the top. (**f–h’’**) Expression of pan-mesodermal marker *brachyury (xbra)*. (**f**) Uninjected St. 10.5 embryo (N = 3; n = 47). (**f’**) St-MO injected St. 10.5 embryo (N = 2; n = 31). (**f’’**) *fry*-MO injected St. 10.5 embryo (N = 3; n = 53). (**g**) Uninjected St. 13 embryo (N = 2; n = 44). (**g’**) St-MO injected St. 13 embryo (N = 2; n = 28; 7% with abnormally positioned expression domain) (**g’’**) *fry*-MO injected St. 13 embryo (N = 2; n = 52; 98% with abnormally positioned expression domain). (**h**) Uninjected St. 15 embryo (N = 3; n = 48). (**h’**) St-MO injected St. 15 embryo (N = 2; n = 33). (**h’’**) *fry*-MO injected St. 15 embryo (N = 3; n = 45). Dorsal views, anterior is oriented to the top. (**i–l’’**) Expression of *not* in the chordamesoderm and anterior neuroectoderm*.* (**i**) Uninjected St. 10.5 embryo (N = 2; n = 52). (**i’**) St-MO injected St. 10.5 embryo (N = 2; n = 31). (**i’’**) *fry*-MO injected St. 10.5 embryo (N = 2; n = 47). (**j**) Uninjected St. 13 embryo (N = 3; n = 39). Dorsal is oriented to the top. (**j’**) St-MO injected St. 13 embryo (N = 2; n = 27; 4% abnormally positioned expression domain). (**j’’**) *fry*-MO injected St. 13 embryo (N = 3; n = 43; 100% with abnormally positioned expression domain). (**k**) Uninjected St. 13.5 embryo (N = 2; n = 29). (**k’**) St-MO injected St. 13.5 embryo (N = 2; n = 24). (**k’’**) *fry*-MO injected St. 13.5 embryo (N = 2; n = 32, 63% with abnormally positioned expression domain). (**l**) Uninjected St. 15 embryo (N = 3; n = 46). (**l’**) St-MO injected St. 15 embryo (N = 2; n = 28). (**l’’**) *fry*-MO injected St. 15 embryo (N = 3; n = 59). Dorsal views, anterior is oriented to the top. Note: *not* expression in the epiphysis (arrowheads) is initiated in *fry*-depleted embryos at the same time as controls, indicating the morphants are not developmentally delayed. Dashed lines indicate dorsal mesoderm elongation. The stage of injected embryos was established based on the stage of uninjected embryos from the same clutch. Representative embryos are shown. Embryos were injected into both dorsal blastomeres at the 4-cell stage with 15 ng of *fry*-MO or St-MO.

Anterior–posterior axis development is the result of early establishment of mesodermal cell fates and morphogenetic movements that contribute to cell rearrangements during gastrulation^1,3,29^. As Fry has roles in mesoderm development^27,28^ and depleted embryos exhibited reduced head structures and a shortened axis^27^, we tested the expression of early genes corresponding to different cell subpopulations of the dorsal organizer. At early gastrula stages, the expression domains of *orthodenticle homeobox 2 (otx2)*^30^ and *goosecoid (gsc)*^31^ genes in the presumptive prechordal mesoderm were reduced in *fry*-MO injected embryos (Fig. 2a–b’’). Similarly, in the presumptive axial mesoderm, the *chordin (chrd)*^32^ expression domain was reduced in *fry*-depleted embryos (Fig. 2d–d’’), while expression of *notochord homeobox (not)*^33^ in the early chordamesoderm appeared unaffected (Fig. 2i–i’’). These findings are in agreement with a previous publication^27^ and suggest that Fry modulates the cell fate of specific populations of the dorsal organizer, particularly cells of the presumptive prechordal mesoderm.

In the absence of Fry, the pan-mesodermal marker *brachyury (xbra)*^34^ was expressed at the gastrula stage (Fig. 2f–f’’). Furthermore, at late tailbud stage, *chrd* in the notochord and *myogenic differentiation 1 (myoD)*^35^ in the somites were both expressed in the absence of Fry (Supplementary Fig. S2a–d). Despite the shortening of the anterior–posterior axis, differentiated notochord and somites (MZ15 and 12/101 antibodies, respectively) were present in both mild and more severe *fry* morphants phenotypes (Supplementary Fig. S2e–h). While both structures appeared morphologically abnormal, differentiation was not severely impaired. Our data indicate that Fry is not involved in general mesoderm induction or expression of terminal differentiation markers of axial mesoderm tissues.

Next, we investigated the effect of Fry loss-of-function in the expression patterns of these genes during *Xenopus* gastrulation. Consistent with gastrulation defects, the blastopore remained opened beyond late gastrula stages (St. 13) in most *fry*-depleted embryos (Fig. 2c’’,e’’,g’’,j’’,k’’). In addition, the expression domains of *chrd* and *gsc* were not only reduced, but abnormally positioned in these embryos (Fig. 2c–c’’,e–e’’). *Chrd-*positive cells marking the axial mesoderm appeared to reflect defects in involution (Fig. 2e’’). Furthermore, the expression domain did not extend along the anterior–posterior axis nor converge mediolaterally to the midline (Fig. 2e–e’’). In addition, movement of *gsc* expressing prechordal mesoderm cells toward the animal pole was impaired in *fry* morphants (Fig. 2c–c’’). The expression patterns of *xbra* and *not* at late gastrula stage were also abnormally positioned reflecting defects in midline elongation in *fry*-depleted embryos (Fig. 2g–g’’,k–k’’). The neuroectodermal *not* expression that initiates at St13.5 indicates that gene expression patterns are not altered in *fry*-MO injected embryos due to developmental delay, but rather reflect defects in gastrulation movements in these embryos. At neurula stage most embryos closed their blastopore, however, the notochord does not extend normally in *fry* depleted embryos (Fig. 2h–h’’,l–l’’). The elongation of dorsal midline tissues was quantified as the length of *not* gene expression domain in early and late-gastrula embryos^36^. As expected, dorsal mesoderm elongation was reduced in the absence of Fry and partially rescued by *FD* + *LZ* mRNA injection (Supplementary Fig. S3). In light of these results, we decided to further characterize the defective morphogenetic movements associated with the loss of Fry function.

### Fry function is necessary for normal blastopore closure

**Figure 3:**
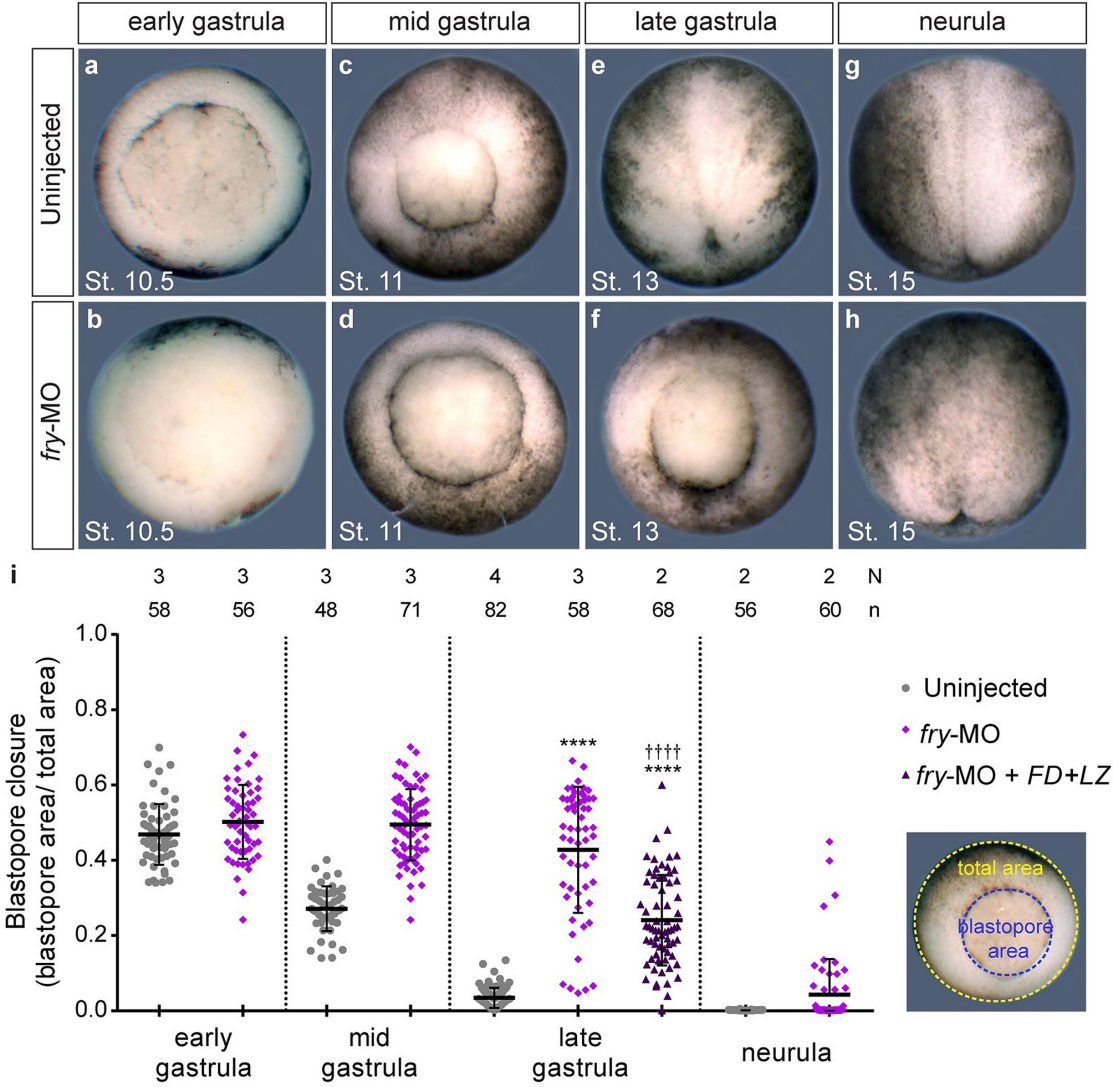
Fry is required for normal blastopore closure. (**a**–**h**) Uninjected or *fry*-MO (15 ng) injected embryos were fixed at the indicated gastrulation stage for blastopore closure measurements. Representative embryos for each stage are shown. Gastrula stage embryos are oriented dorsal to the top and neurula stage embryos are oriented anterior to the top. (**i**) Left: Quantification of the blastopore closure measurements on fixed uninjected embryos, *fry*-MO (15 ng) injected embryos and *fry*-MO (15 ng) + *FD* + *LZ* mRNA (800 pg) co-injected embryos at the indicated gastrulation stage. Right: Image showing the blastopore area (blue) and the area of the vegetal hemisphere of the embryo (total area, yellow). The ratio of these two measurements is plotted. 0 = blastopore closed. N: number of independent experiments, n: number of embryos. The stage of injected embryos was established based on the stage of uninjected control embryos. Means and standard deviation are indicated. Each point represents a single fixed embryo. Statistical significance was evaluated using Kruskal–Wallis test and Dunn's multiple comparisons test (****,^††††^*p* < 0.0001). * represents the comparison to the uninjected group and † represents the comparison to the *fry*-MO injected group.

Blastopore closure is a major contributor to gastrulation in amphibians involving the coordination of multiple morphogenetic movements in the embryo^3,37^. To assess blastopore closure progression from the initial step of blastopore formation at the early gastrula until its closure at the early neurula, we measured the blastopore area on fixed embryos at different stages (Fig. 3a–h). While most uninjected embryos closed their blastopore by late gastrula stage, the blastopore remained open in most of *fry*-depleted embryos at this stage. Yet, by mid-neurulation, all embryos close their blastopore with the exclusion of some with extruding yolk plugs (25%), suggesting either a non-specific developmental delay or a specific disruption of dorsal morphogenesis (Fig. 3i). Confirming the rescue of the *fry* morphant phenotype observed at the tailbud stage by co-injection of *FD* + *LZ* mRNA (Fig. 1), the blastopore closure defect induced by *fry* knock-down was also partially rescued by *FD* + *LZ* (Fig. 3i).

The rescue of the *fry* knock-down argued against a non-specific delay, so we sought to further characterize the dynamics of blastopore closure. We acquired time-lapse sequences of gastrulating embryos from the onset of gastrulation until blastopore closure (Supplementary Movie S1). In these time-lapses (n = 12) we observed that blastopore formation, which is initially restricted to a small region on the dorsal side of the uninjected embryo, was laterally expanded in most *fry* morphants (10/12 embryos) (yellow dotted arrows, Supplementary Fig. S4). We saw a complete yolk-encircling ring of bottle cells forming in both groups of embryos. Additionally, by late gastrula stages, most *fry*-depleted embryos closed their blastopore concentrically (9/12 embryos) as opposed to the characteristic dorsal-dominated eccentric closure of the blastopore toward the ventral side (see Supplementary Movie S1 and Supplementary Fig. S4). Hence, Fry depletion in dorsal tissues alters blastopore formation and the dynamics of blastopore closure, but bottle cell formation appears coincident with control embryos.

### *Fry* depletion affects movement of superficial involuting marginal zone cells during gastrulation

**Figure 4:**
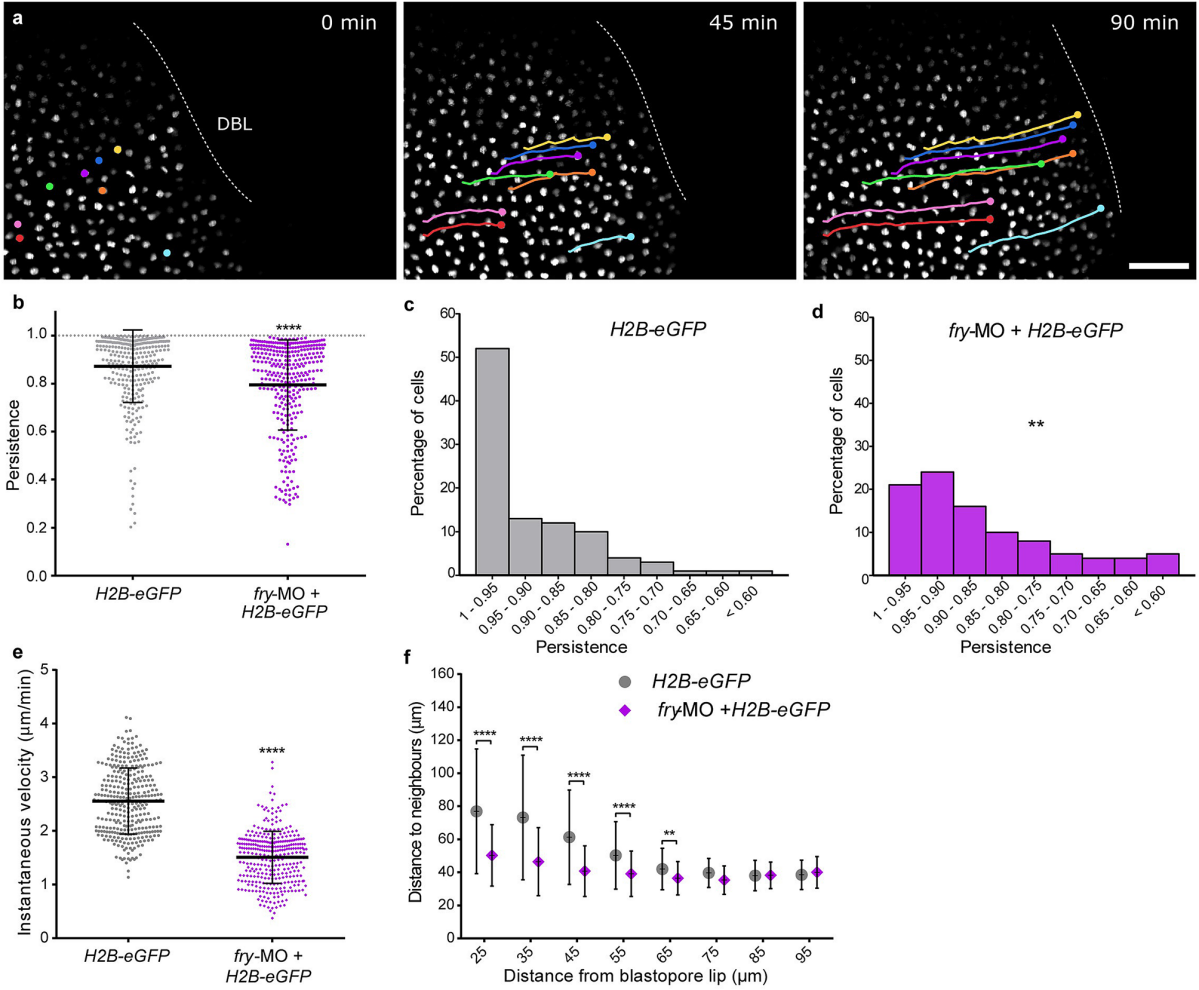
Fry depletion affects the motion of superficial involuting marginal zone cells. (**a**) *Xenopus* 4-cell stage embryos were dorsally injected with *H2B-eGFP* mRNA with or without *fry*-MO (15 ng) and mounted for *light-sheet* fluorescence microscopy at the beginning of gastrulation (St. 10). Time lapse movies were recorded during gastrulation and individual cells were tracked while moving toward the dorsal blastopore lip (DBL) (e.g. nuclei in color. Circles and lines indicate nuclei position and trajectory, respectively). *H2B-eGFP* mRNA (N = 5; total number of tracked cells = 319); *H2B-eGFP* mRNA + *fry*-MO injected embryos (N = 5; total number of tracked cells = 332). N: number of independent experiments. Representative images from time-lapse movies at selected time-points (t) are shown. Scale bar: 100 μm. (**b**) Individual cell persistence measurements were calculated as the ratio between the linear distance traveled by the cell and the total length of its path. Each point represents a single cell. Statistical significance was evaluated using two-tailed Mann Whitney *U*-test. ****p < 0.0001 indicates statistically significant differences between groups. The mean and standard deviation are indicated. (**c**) Histogram representing the percentage of cells from control embryos (*H2B-eGFP*) for the different persistence intervals. (**d**) Histogram representing the percentage of cells from *fry-*MO + *H2B-eGFP* embryos for the different persistence intervals. Statistical significance was evaluated using *Chi*-square test. **p < 0.001 indicates statistically significant differences were found between groups. (**e**) Individual cell instantaneous velocity measurement. The mean and standard deviation are indicated. Each point represents a single cell. Statistical significance was evaluated using two-tailed Mann Whitney *U*-test. ****p < 0.0001 indicates statistically significant differences between groups. (**f**) Average distance from each cell nucleus to the nearest neighbors for all cells within a certain distance region from the blastopore lip (region size = 30 μm; overlapping region size = 5 μm). Distance to neighbors was quantified from the 150 min time point onward (St. 11.5) of the movies shown in a. Number of cells in each window was always larger than 100 cells. Data in the graph is presented as means with standard deviation. Statistical significance was evaluated using Kruskal–Wallis test and Dunn's multiple comparisons test. ****p < 0.0001 and **p < 0.01 indicate statistically significant differences between groups.

Since blastopore closure is driven by multiple processes including convergent thickening, convergent extension, and involution^2,37^, we first investigated the impact of Fry depletion on the motion of the superficial cells during involution^38^. To achieve this, we injected dorsal blastomeres with *H2B-eGFP* mRNA with or without *fry*-MO and mounted the embryos for *light-sheet* fluorescence microscopy. This technique allowed us to visualize and track dorsal cell nuclei over several hours with negligible levels of photobleaching in intact, live gastrulating embryos^39,40^ (Supplementary Movie S2) (Fig. 4a). As a measure of motion directionality, we evaluated the persistence of each cell as it moved towards the DBL. Persistence was defined as the ratio between the linear distance traveled by a cell and the total length of its path: cells that move in a straight line will have persistence of 1, whereas cells that move in a more erratic trajectory will have lower persistence. We observed that Fry depletion significantly reduced directional persistence with a mean of 0.93 ± 0.06 for cells of control embryos versus a mean of 0.85 ± 0.05 for cells of *fry*-depleted embryos (Fig. 4b). For control embryos, the majority of the cells (> 50%) had persistence values between 1 and 0.95 while very few cells had values below 0.70. By contrast, for *fry*-depleted embryos, fewer than 20% of the cells fall within the 1–0.95 persistence interval while the remaining 80% had lower persistence values (Fig. 4c). These results indicate that cells trajectories towards the DBL are severely affected in the absence of Fry (Fig. 4c,d). Next, we defined instantaneous velocity as the spatial displacement over time for two consecutive frames. In consistency with the observed blastopore closure delay, Fry depletion significantly reduced the instantaneous velocity of individual cells as they move towards the DBL (Fig. 4e).

To investigate whether the spatial organization of cells on the superficial involuting marginal zone (IMZ) was affected in *fry*-depleted embryos as a result of the observed velocity and persistence changes, we estimated the distance between nearby cell nuclei as a function of the distance from the DBL (see Methods). While cells 75–95 μm from the DBL showed a similar distance to neighboring cells in control and *fry*-depleted embryos, cells closer to the DBL (up to 65 μm) presented a significant reduction of the distance to neighbors in *fry* morphants (Fig. 4f). This result reveals an alteration of the geometrical organization in the pre-involution zone (up to 30 μm from the DBL) of the superficial IMZ as a result of Fry depletion. The observed increase of cell density or “bunching” in the proximity of the involution site in *fry-*depleted embryos, could indicate that while superficial IMZ cells are experiencing convergence forces, they are not being pulled over the blastopore lip by forces generated by post-involution tissues. These results demonstrate a dorsal-requirement for Fry during involution movements and suggest that convergent thickening might be operating in *fry* morphants allowing blastopore closure, even under conditions where convergent extension is likely defective^2,37^.

### Fry function is necessary for formation of the cleft of Brachet and the associated fibronectin network

At the start of gastrulation, a deep cleft named the cleft of Brachet forms around the marginal zone, separating mesendoderm from prospective pre-involution mesoderm and non-involuting ectoderm. The cleft is thought to form through changes in cell adhesion and vegetal rotation^41,42^. Leading-edge mesendodermal cells on one side of the cleft contact and move along the ectoderm blastocoel roof (BCR) on the other side of the cleft. The interface becomes the site where an ECM rich in fibrillar fibronectin is assembled that is essential for mesendoderm migration^43,44^. As mesodermal cells (first prechordal mesoderm and later chordamesoderm) move over the base of the cleft, the so-called inner lip, post-involution cells acquire the capacity to remain separated from the pre-involution and non-involuting ectodermal cells.

**Figure 5:**
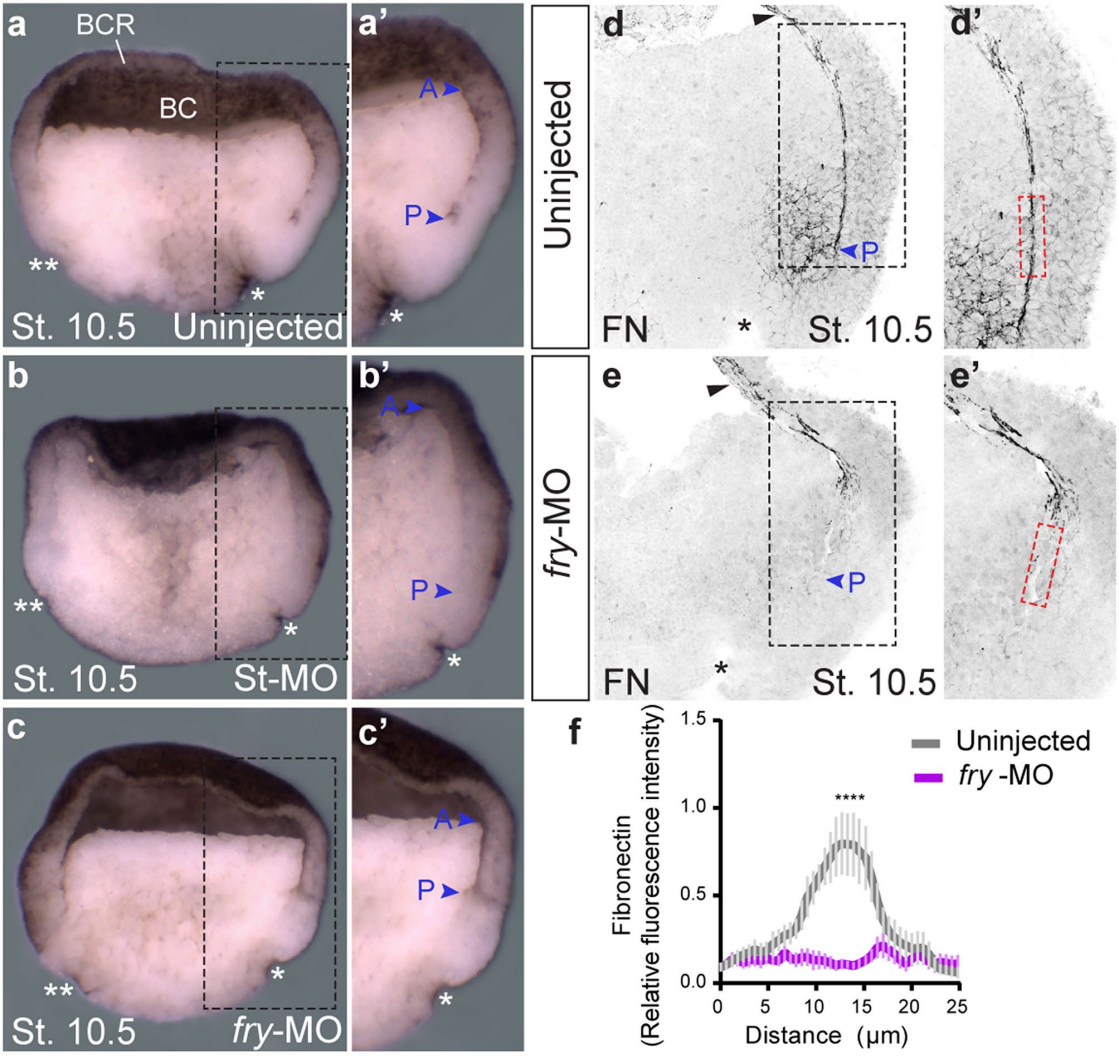
The cleft of Brachet and fibronectin fibrillar matrix formation are affected in *fry*-depleted embryos. (**a–c**) Formation of the cleft of Brachet on the dorsal side was analyzed in hemisected early gastrula stage embryos (St. 10.5). (**a**) Uninjected embryo (N = 2, n = 26). (**b**) Standard Control morpholino (St-MO) (15 ng) injected embryo (N = 2; n = 13). (**c**) *fry*-MO (15 ng) injected embryo (N = 2; n = 25, in 92% of embryos, the cleft of Brachet is only present anteriorly). * indicates the position of the dorsal blastopore lip; ** indicates the ventral blastopore lip (morphological feature used as indication that embryos were developmentally synchronized at stage 10.5); blue arrowheads indicate the anterior (A) and posterior ends (P) of the cleft, BC: blastocoel; BCR: blastocoel roof. (**a’–c’**) Magnifications of the black-boxed area in panels a-c. Representative embryos are shown. (**d,e**) Hemisections of early gastrula stage embryos (St. 10.5) immunostained for fibronectin. (**d**) Uninjected embryo. (**e**) *fry*-MO (15 ng) injected embryo. Black arrowheads indicate fibronectin presence at the BCR. Blue arrowheads indicate the posterior end (P) of the cleft. (**d’,e’**) Magnifications of the black-boxed area in panels d and e. For fibronectin quantification, a rectangle 25 μm wide and 100 μm long was drawn at a distance of 300 μm from the dorsal blastopore lip (*) across the cleft of Brachet (red-boxed area). (**f**) Fibronectin abundance was quantified as fluorescence intensity across the 25 μm width of the red rectangle and normalized to the mean fluorescence (see Methods) in uninjected embryos (N = 2; n = 7) and *fry*-MO (15 ng) injected embryos (N = 2; n = 8). Data in the graph is presented as mean with standard error. Statistical significance was evaluated using two-tailed Mann Whitney *U*-test. **** *p* < 0.0001 indicates statistically significant differences between groups.

To assess the role of Fry in the formation of the cleft of Brachet, we analyzed hemisected fixed *fry* morphant and control embryos. In *fry*-depleted embryos, we found that while the cleft of Brachet was detected anteriorly, the boundary between the involuted mesoderm and pre-involuting cells was not observed near the blastopore lip (Fig. 5a–c and insets a’–c’). Given that abnormalities in the formation of the cleft of Brachet are frequently associated with tissue separation defects, we evaluated this behavior using ectoderm/BCR assays^44,45^. Involuted mesoderm test aggregates of uninjected and *fry*-MO injected embryos remained on the explanted BCR surface, demonstrating that internalized mesodermal cells are capable of maintaining separation from the explanted BCR cells (Supplementary Fig. S5). As expected, aggregates of uninjected and *fry*-MO injected embryos isolated from the inner layer of the BCR merged into the explanted BCR surface, as those cells do not express separation behavior (Supplementary Fig. S5). Finally, as the cleft of Brachet is later stabilized by the assembly of a fibronectin fibrillar matrix^44^, we investigated its formation in early gastrula *fry*-depleted embryos. We observed that the normally abundant fibronectin fibrils that form in the cleft were reduced (Fig. 5d–f and insets d’,e’), while other populations of fibrils on the BCR were not affected (Fig. 5d,e black arrowheads).

Taken together, our findings indicate that while the formation of the posterior-most domain of the cleft appears affected by loss of Fry function, post-involution cells have the ability to remain separated from non-involuted ectodermal cells measured by BCR assays. Further, we suspect that in *fry* morphants, the failure of fibronectin matrix assembly alters the stability of the cleft.

### Fry is required for elongation and mediolateral orientation of dorsal mesoderm cells

**Figure 6:**
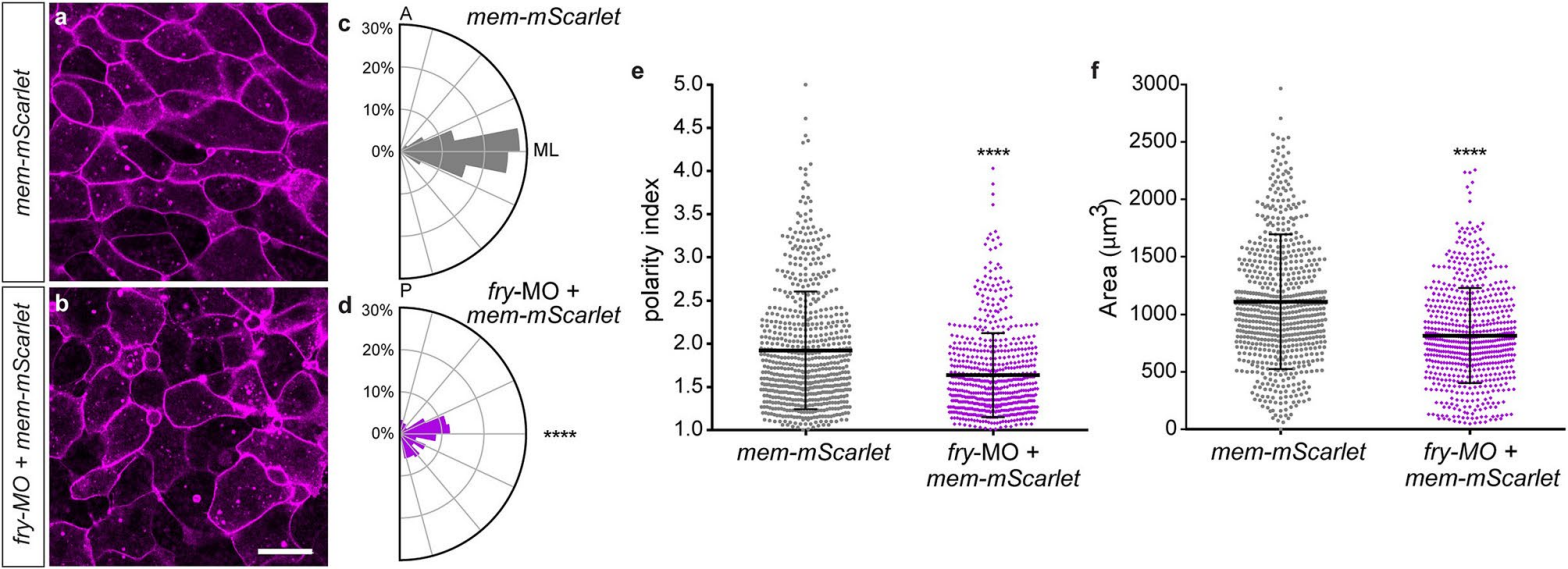
Loss of Fry affects morphological polarity and orientation of dorsal mesoderm cells. (**a,b**) Dorsal marginal zone explants were prepared from *Xenopus* embryos at early gastrula stage (St. 10.5) and the vegetal alignment zone (VgAZ)^5^, which corresponds to the axial mesoderm, was imaged at late gastrula stage (St. 13) to assess dorsal mesoderm cell morphology. (**a**) *mem-mScarlet* mRNA injected embryo (N = 2, n = 7). (**b**) *fry-*MO (15 ng) + *mem-mScarlet* mRNA co-injected embryo (N = 2, n = 7). N: number of independent experiments, n: number of explants. Scale bar: 100 μm. Representative explants are shown. (**c**,**d**) Cell orientation was quantified as the angle of the cell’s major axis with respect to the mediolateral axis (ML). The circles in the rose diagram refer to the percentage of cells that exhibited polarity angles for each bin. Orientation angles were binned from 0° to 90° in bins of 11.25°. A: anterior, P: posterior, ML: mediolateral. (**c**) *mem-mScarlet* mRNA injected embryo (total number of cells analyzed = 147). (**d**) *fry-*MO + *mem-mScarlet* mRNA co-injected embryo (total number of cells analyzed = 151). Statistically significant differences were found between groups, *Chi*-square test (****p < 0.0001). (**e**) Polarity index measurements of dorsal mesoderm cells calculated as the ratio between cell major axis and minor axis. (**f**) Cellular area measurements of dorsal mesoderm cells. (**e,f**) *mem-mScarlet* mRNA injected embryos (total number of cells analyzed = 660); *fry*-MO + *mem-mScarlet* mRNA co-injected embryos (total number of cells analyzed = 632). Each point represents a single cell. Statistical significance was evaluated using two-tailed Mann Whitney *U*-test. **** *p* < 0.0001 indicates statistically significant differences between groups. Means and standard deviation are indicated.

Convergent extension within dorsal midline tissues extends the body axis and aids blastopore closure^5,37^. To drive convergent extension, mesoderm cells must undergo directed cell rearrangement through mediolateral cell intercalation, a cell behavior marked by mediolateral cell elongation and alignment, called mediolateral intercalation behavior (MIB)^46^. To evaluate whether Fry plays a role in regulating the MIB, we assessed cell shape and orientation in DMZ explants isolated from embryos dorsally injected with *fry*-MO and the mRNA of a membrane marker (mem-mScarlet)^47^. We quantified the orientation of dorsal mesoderm cells within the explants by plotting the cell’s major angle with respect to the mediolateral axis on a rose diagram. Mesodermal cells within *mem-mScarlet* injected explants orient along the mediolateral axis on fibronectin-coated substrate (Fig. 6a,c). By contrast, mesodermal cells in explants injected with *fry*-MO under the same culture conditions fail to orient (Fig. 6b,d). The degree of cell shape polarization or “polarity index” of dorsal mesoderm cells was measured as the ratio between cell major and minor axes^48,49^. Cells lacking Fry exhibit a lower polarity index (1.64 ± 0.02) relative to cells from embryos injected with the mRNA alone (1.92 ± 0.03) (Fig. 6e). These experiments demonstrate that Fry function is required for the expression of MIB in the dorsal mesoderm. In the absence of Fry, dorsal mesoderm cells presented a smaller area than cells in the control group evidencing additional changes in cell shape (Fig. 6f).

### Human NDR1 kinase partially rescues axis elongation of *fry*-depleted embryos

In mammalian cells and invertebrates, Fry orthologs genetically and physically interact with NDR kinases regulating their activity^14–16,18,21,24,26^. We decided to investigate whether NDR1 kinase plays a role downstream of Fry in anterior–posterior axis formation and the morphogenetic events of gastrulation. To this end, we overexpressed a *wild-type* version of human NDR1 (hNDR1-wt), a constitutively active form named hNDR1-PIF^50^, which mimics an active kinase, and a kinase-dead version named hNDR1-kd^51^. Dorsal injection of all *hNDR1* forms caused reduction of head structures and mild axis elongation defects in ~ 50% of the embryos (Supplementary Fig. S7a–d). These results indicate that dorsal overexpression of all three human NDR1 functional variants affects anterior–posterior axis development in *Xenopus*. Additionally, we evaluated the ability of *hNDR1* variants overexpressed in ventral blastomeres to induce secondary axes, as this capacity has been reported for *fry* ventral overexpression^27^. In agreement with a previous report by Goto et. al.,^27^ *hNDR1-wt* had no effect on axis or head formation (Supplementary Fig. S7e). Similarly, neither *hNDR1-PIF* nor *hNDR1-kd* ventral overexpression affected axis formation or generated an ectopic axis (Supplementary Fig. S7f,g).

**Figure 7:**
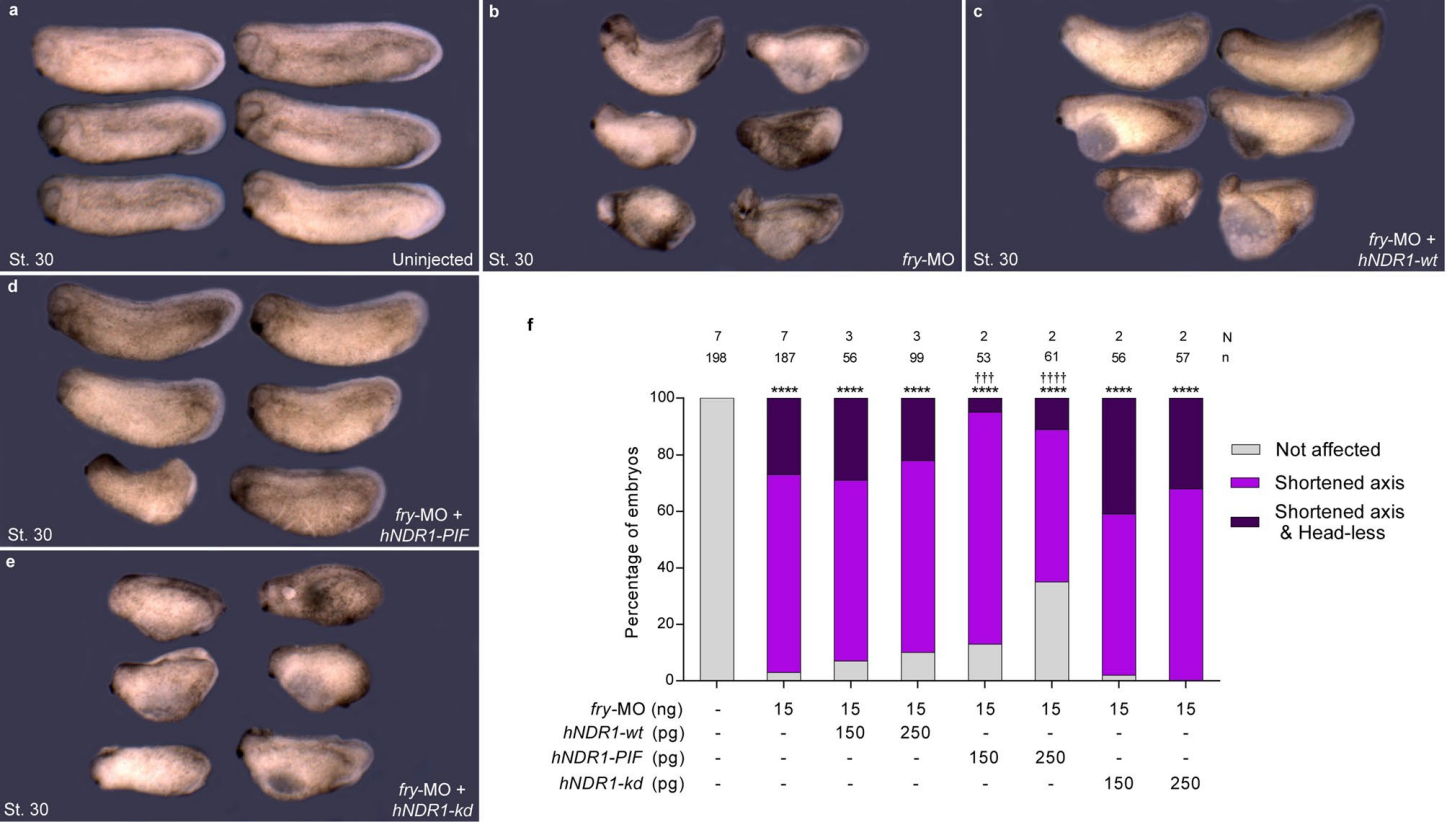
hNDR1-PIF partially rescues axis elongation in *fry*-depleted embryos. (**a–e**) 4-cell *Xenopus* embryos were injected into both dorsal blastomeres as indicated and fixed at St. 30. (**a**) Uninjected embryos. (**b**) *fry*-MO (15 ng) injected embryos. (**c**) *fry*-MO (15 ng) + *hNDR1-wt* mRNA (250 pg) co-injected embryos (**d**) *fry*-MO (15 ng) + *hNDR1-PIF* mRNA (250 pg) co-injected embryos. (**e**) *fry*-MO (15 ng) + *hNDR1-kd* mRNA (250 pg) co-injected embryos. Representative embryos are shown. (**f**) Quantitation of the percentage of embryos showing the different phenotypes: “Not affected”, “Shortened axis” or “Shortened axis & Head-less” phenotypes. Embryos were scored as “Shortened axis” when presented < 80% of the body length relative to the average length of control embryos. Data on graph is presented as mean. N: number of independent experiments, n: number of embryos. Statistical significance was evaluated using *Chi*-square test (****,^††††^*p* < 0.0001 and ^†††^*p* < 0.001). * represents the comparison to the uninjected group and † represents the comparison to the *fry*-MO injected group.

To evaluate Fry and NDR1 functional interaction in axis elongation and development of anterior structures, we performed injections of the different *hNDR1* mRNAs individually or with *fry*-MO. Dorsal co-injection of *hNDR1-wt* or *hNDR1-kd* failed to rescue Fry depletion (Fig. 7b,c,e,f), while co-injection of *hNDR1-PIF* mRNA resulted in a significant suppression of the tailbud *fry* morphant phenotype (Fig. 7d,f). Although most embryos co-injected with *hNDR1-PIF* mRNA and *fry-*MO presented axis elongation defects and therefore scored as “shortened axis”, < 80% body length relative to the average length of control embryos, the truncation of the axis was clearly less severe in these embryos relative to *fry* morphants (Fig. 7b,d and see Supplementary Table S1).

### hNDR1-PIF partially rescues impaired convergent extension in *fry*-depleted embryos

**Figure 8:**
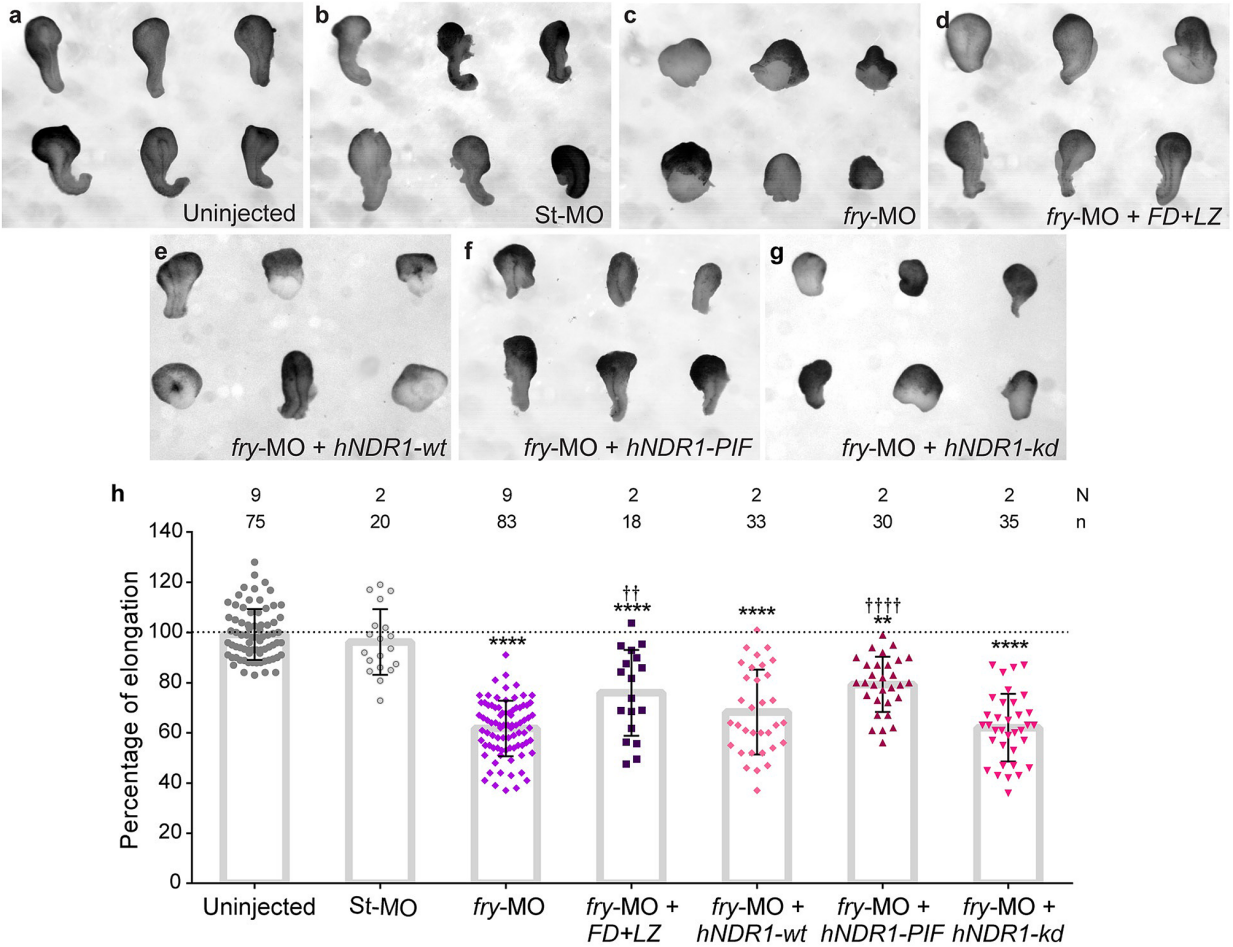
Loss of Fry impairs convergent extension movements and can be compensated by constitutively active hNDR1-PIF kinase**.** (**a**–**g**) Dorsal marginal zone explants were prepared from *Xenopus* embryos at early gastrula stage (St. 10.5) and culture until late neurula stage (St. 19) when the elongation of the explant was evaluated. (**a**) Uninjected embryos. (**b**) Standard Control morpholino (St-MO) (15 ng) injected embryos. (**c**) *fry*-MO (15 ng) injected embryos and (**d**) *fry*-MO (15 ng) + *FD* + *LZ* mRNA (800 pg) co-injected embryos. (**e**) *fry*-MO (15 ng) + *hNDR1-wt* mRNA (250 pg) co-injected embryos (**f**) *fry*-MO (15 ng) + *hNDR1-PIF* mRNA (250 pg) co-injected embryos and (**g**) *fry*-MO (15 ng) + *hNDR1-kd* mRNA (250 pg) co-injected embryos. Representative explants are shown. (**h**) Percentage of elongation of dorsal marginal zone explants from embryos treated as indicated. Elongation was calculated as the difference between the initial and final length of the explants (St. 10.5 vs. St. 19) relative to the mean of the uninjected group (considered 100% elongation, dotted line). Each point represents a single explant. N: number of independent experiments, n: number of explants. Statistical significance was evaluated using Kruskal–Wallis test and Dunn's multiple comparisons test (****,^††††^*p* < 0.0001 and **,^††^*p* < 0.01). * represents the comparison to the uninjected group and † represents the comparison to the *fry*-MO injected group.

To address whether the defective MIB exhibited by *fry*-depleted cells results in impaired convergent extension, we analyzed the elongation of DMZ explants^52^. Unlike control explants that elongate as a result of convergent extension (Fig. 8a,b), elongation of DMZ explants from *fry*-depleted embryos was strongly inhibited (Fig. 8c,h). Additionally, coinjection of *FD* + *LZ* mRNA along with *fry*-MO significantly restored explant elongation (Fig. 8d,h), consistent with the axis elongation rescue at tailbud stage (Fig. 1). These results indicate that defective convergent extension is, at least in part, responsible for the shortened axis phenotype.

Next, we evaluated whether the different functional variants of hNDR1 were able to rescue convergent extension in *fry*-depleted embryos. The analysis of DMZ explants elongation showed that *hNDR1-PIF* partially compensates for Fry loss-of-function (Fig. 8f,h). However, when *hNDR1-wt* or *hNDR1-kd* were co-injected with *fry*-MO, explants elongated to a small degree, but the differences with explants from embryos injected only with *fry*-MO were not significant (Fig. 8e,g,h). Complementary to these results, we quantified dorsal mesoderm elongation in intact neurula-stage embryos by measuring the length of the *not* expression domain. We observed a modest but significant rescue when co-injecting *hNDR1-PIF* along with *fry*-MO (Supplementary Fig. S6)*.* Together, these results show that in the absence of Fry function, only constitutively active hNDR1 is able to rescue convergent extension, suggesting that kinase activity is required. The lack of significant rescue by *hNDR1-wt* further suggests that activation of this kinase still requires Fry function.

Our data are in agreement with previous findings showing that Fry functionally interacts with NDR1 kinase, and support our hypothesis that Fry function in dorsal axis elongation and convergent extension is mediated, at least in part, by the activation of NDR1 kinase.

## Discussion

Despite the fact that Fry is an evolutionarily conserved protein from yeast to humans, very little is known about its function. At the beginning of gastrulation, *fry* transcripts are present in the marginal zones supporting a role in the initial gastrulation movements. We found that Fry loss-of-function consistently affects the movements and spatial configuration of the superficial and deep IMZ cells. As gastrulation proceeds, *fry* mRNA is enriched in the axial and paraxial mesoderm and the deep ectodermal layer, all tissues that undergo convergent extension. In this regard, our studies demonstrate a requirement of Fry in regulating cell behaviors that underlie morphogenetic movements of midline tissues.

During amphibians gastrulation, two intrinsic convergence behaviors of the IMZ contribute to blastopore closure: convergent thickening and convergent extension. While convergent extension is exclusively conducted by the presumptive dorsal tissues of the mid-gastrula, convergent thickening operates in all pre-involuting circumblastoporal tissues in a symmetric fashion. Moderate dorsal patterning defects have little impact on convergent extension as embryos lacking a notochord can form tadpoles that are indistinguishable from *wild-type*^53,54^. As it was demonstrated in severely UV ventralized embryos, convergent thickening can drive blastopore closure on its own in the absence of convergent extension^37,55,56^. We observed that, similar to severely ventralized embryos, dorsal Fry depletion alters the initial blastopore formation and blastopore closure dynamics, suggesting that convergent thickening still operates in the absence of Fry. Additionally, our cell tracking recordings of *fry*-depleted embryos reveal alterations in the movement of superficial IMZ cells and the spatial organization of the cells near the blastopore lip, revealing a lack of coordination between the forces that operate in the system.

The fibronectin component of the ECM plays a critical role in the regulation and maintenance of gastrulation movements and blastopore closure^48^. Fibronectin in the cleft of Brachet provides physical support for mesendoderm cell migration^43,57,58^, as well as a source of signaling^59,60^. Loss of fibronectin can lead to alterations in the migratory kinetics of leading-edge mesodermal cells^48^ and therefore might also contribute to the abnormal development of anterior structures in *fry*-depleted embryos^29,61^. As in fibronectin knock-down embryos, convergent extension is disrupted in *fry*-depleted embryos. However, DMZ cells lacking Fry fail to elongate and orient when provided an exogenously fibronectin substrate, suggesting the existence of an additional mechanism affecting the morphogenetic behavior of these cells. Based on these migratory defects and loss of cell polarization and orientation, it is possible that Fry regulates cytoskeleton dynamics. In *Drosophila,* Fry has been found to alter actin organization during egg chamber elongation and hair wing morphogenesis, as well as to regulate microtubule sliding in neurons^13,15,62^. Given that Fry binds to microtubules and regulates their dynamics in *Drosophila* and mammalian cells^21–23^, controlling chromosome alignment, mitotic spindle orientation and morphogenesis, it is also possible that defects in cell division could contribute to the phenotype of *fry* morphants. Whether Fry regulates cytoskeletal components involved in morphogenesis and cell division remains to be determined. Fry also promotes dendrite attachment to the ECM in *Drosophila* dendritic arborization neurons, but the mechanism remains elusive^63^. Whether Fry is required for intercellular adhesion or cell-ECM adhesion via integrins in our system, will require further investigation.

Convergent extension is under the control of multiple molecular pathways^64^, among them, importantly, the non-canonical Wnt/PCP signaling pathway^4,65–69^. In addition to regulating cell polarity and the coordination of morphogenetic behaviors, the PCP pathway regulates polarized ECM deposition required for convergent extension^66,70^. Many characteristics of the Fry loss-of-function phenotype, such as blastopore closure defects, convergent extension impairment, dorsal mesoderm MIB disruption, and the reduction of the fibronectin fibrillar matrix assembly along the mesoderm surface, resemble PCP pathway perturbations^66^. While evidence of genetic interactions between PCP components and the Fry-Tricornered pathway (Tricornered, *Drosophila* ortholog of NDR1) have been described for dendritic self-avoidance^71^, further studies will be required to determine whether there is physical and/or functional interaction between Fry and the PCP signaling pathway in *Xenopus*.

Previous studies, and our findings suggest the involvement of Fry function in early cell fate decisions in *Xenopus* regulating anterior specification. The development of head abnormalities in the *fry* morphants could likely be attributed to early prechordal mesoderm genes misexpression. However, as it was shown in prior studies of PCP mutants, defects in anterior patterning can arise when patterns of cell motility are disrupted^72^. In this sense, it will have to be determined whether the morphogenetic defects associated with Fry loss-of-function have an impact on early patterning.

Multiple studies in invertebrates and ours here in *Xenopus* point to a role of Fry protein in cellular mechanisms driving morphogenesis. Fry was originally identified in *Drosophila melanogaster* where its mutation causes disorganized epidermal cell morphology^13^. A very similar phenotype found in *tricornered* gene mutants suggested that these proteins physically interact and function in a common pathway^13,14,18^. Our rescue experiments show that human constitutively active NDR1, in contrast to the *wild-type* and kinase-dead variants, can partially compensate for the loss of Fry function, suggesting that Fry is necessary for NDR activation during axis development in *Xenopus*. Our results argue in favor of a conserved functional interaction between Fry and NDR kinases in animal development. The partial rescue of Fry-depletion phenotypes by NDR1 suggests that this kinase might not be the only protein mediating Fry function in these processes. In fact, Fry appears to have NDR-independent functions in mammalian cells^22–24^ and in nematodes^16^. Moreover, in *Xenopus* embryos, ventral overexpression of Fry induces a secondary axis, while it has been shown here and in a previous work^27^ that NDR1 does not have this capacity. The specific function of *Xenopus* Stk38 (*stk38*; orthologue of human NDR1) in axis elongation and convergent extension movements will require further investigation. Further, the precise mechanism by which overexpression of hNDR1 functional variants lead to an abnormal axis development will have to be determined. Similarities between the phenotypes associated with loss-of-function and gain-of-function are not uncommon to the events that regulate gastrulation movements^11,68,73^. We can speculate that hNDR1-kd is acting in a dominant-negative fashion by sequestering necessary interacting proteins. Alternatively, the observed phenotype could be explained by a kinase-independent mechanism resulting in a gain-of-function. Discrimination between these possibilities will require further analysis and exceeds the scope of this work.

In the present work, we show for the first time that Fry is a regulator of cell movements and morphogenesis during gastrulation. Genetic studies in yeast, nematode and fruit fly have revealed a critical role of Fry in morphogenesis and cell polarity associated with NDR activation; however, the molecular and cellular mechanisms are not well understood. Future research on the identification of additional upstream and downstream factors will provide important insight into the mechanism of action of Fry.

## Materials and methods

### Ethics statement

This study was carried out in strict accordance with the recommendations in the Guide for the Care and Use of Laboratory Animals of the NIH and also the ARRIVE guidelines. The animal care protocol was approved by the Comisión Institucional para el Cuidado y Uso de Animales de Laboratorio (CICUAL) of the School of Applied and Natural Sciences, University of Buenos Aires, Argentina (Protocol #64).

### Xenopus embryo preparation

*Xenopus laevis* embryos were obtained by natural mating. Adult frogs reproductive behavior was induced by injection of human chorionic gonadotropin hormone. Eggs were collected, de-jellied in 3% cysteine (pH 8.0), maintained in 0.1 X Marc's Modified Ringer's (MMR) solution and staged according to Nieuwkoop and Faber^74^. The embryos were placed in 3% ficoll prepared in 1 X MMR for microinjection.

### Constructs for mRNA synthesis

Human NDR1 constructs were generously provided by Alexander Hergovich. The hNDR1-PIF (constitutively active), hNDR1-wt (wild-type) and hNDR1-kd (kinase-dead)^50,75^ cDNAs were excised from pcDNA3.HA.hNDR1-PIF, DNA3.HA.hNDR1-wt and pcDNA3.HA.hNDR1-kd (K118A) by *BamH*I and *Xho*I digestion and cloned into pCS2 + . The Fry-GFP construct was generously provided by Toshiyasu Goto^27^. Constructs for pCS2 + .H2B-eGFP^76^ and pCS2 + .HA.FD + LZ^28^ have been described previously. pCS2 + .mem-mScarlet was generated using mScarlet^77^ cloned into pCS2 + with a membrane-targeting domain (mem) corresponding to the farnesylation motif from human HRas.

### Morpholino and mRNA microinjections

Capped mRNAs for *fry-GFP*, *mem-mScarlet, H2B-eGFP*, *HA.FD* + *LZ*, *hNDR1-PIF, hNDR1-wt* and *hNDR1-kd* were transcribed in vitro using the mMessage mMachine kit (Ambion) following linearization with *Not*I. Fry morpholino *(fry*-MO) (Gene Tools, LLC) sequence and specificity have been previously published^27^. A Morpholino Standard Control oligo (St-MO) was used as a negative control (Gene Tools, LLC). Morpholinos (MO) or mRNAs were injected into both dorsal blastomeres of 4-cell embryos targeting the DMZ. *Fry*-MO was injected at 5–15 ng per embryo and St-MO at 15 ng per embryo. The doses of injected mRNAs per embryo were as follows: *fry-GFP* (1.2 ng), *mem-mScarlet* (125 pg), *HA.FD* + *LZ* (200–800 pg), *H2B-eGFP* (500 pg), *hNDR1-PIF, hNDR1-wt* and *hNDR1-kd* (150–250 pg).

### In situ hybridization and immunostaining

Whole-mount in situ hybridization was carried out as previously described^78^. *Fry* (Dharmacon), *chordin* (gift from Edward De Robertis) and the *myoD* constructs (gift from Oliver Wessely) were linearized as previously described^28,32,79^. The *Brachyury* construct (gift from Neil Hukriede) was linearized with *Bgl*II, the *not* construct (gift from David Kimelman) was linearized with *Hind*III, the *gsc* construct (gift from Neil Hukriede) was linearized with *EcoR*I, and the *otx2* construct (gift from Ira Blitz) was linearized with *EcoR*I. All linearized constructs were transcribed with T7 for antisense probe synthesis with the exception of *gsc* which was transcribed with SP6. For whole-mount immunostaining with MZ15 (DSHB Cat# MZ15, RRID:AB_760352) and 12/101 (DSHB Cat# 12/101, RRID:AB_531892), we followed the protocol previously described^79^. Fibronectin immunostaining with 4H2 (DSHB Cat# 4H2, RRID:AB_2721949) and subsequent clearing and mounting for confocal microscopy was performed as previously described^43^. Hemisections were cut before staining and imaged with an Olympus FV confocal microscope. Fibronectin immunostaining was measured with ImageJ software (https://fiji.sc/). A 25 μm width and 100 μm length rectangle was drawn around the cleft of Brachet 300 μm away from the DBL. Fluorescence intensity was quantified across the 25 μm width (*I*_*x*_) and normalized to the mean intensity (*I*_*m*_) in order to compare between independent experiments. Fibronectin abundance was plotted as the relative fluorescence intensity *(I*_*x*_ / *I*_*m*_) across the 25 μm width. For preparation of histological slides, embryos processed for in situ hybridization or immunostaining were post-fixed in Bouin’s solution, dehydrated, cleared in xylene, embedded in paraffin and sectioned at 15 μm^28^. Xenbase (http://www.xenbase.org/, RRID:SCR_003280) was used as source of information on gene expression, developmental stages and anatomy.

### Image analysis

Images of fixed whole embryos were collected with a Leica DFC420 camera attached to a Leica L2 stereoscope. Histological slides were imaged using a digital camera (Infinity 1; Lumera Corporation) attached to a light-field microscope (CX31: Olympus). For gastrulation time-lapse sequences, late blastula (St. 9) uninjected and *fry-MO* injected embryos were selected and transferred to custom acrylic chamber with 0.1 X MMR. Gastrulation was recorded for approximately 13 h at 18 °C. Images were taken every 3 min using an Imaging Source SMK 23G445 camera attached to a Zeiss Axiovert S 100 scope. Morphology of gastrulating embryos was quantified from fixed embryos using ImageJ software (https://fiji.sc/). Blastopore closure was calculated as the ratio of the blastopore area over the area of the vegetal hemisphere of the embryo. Elongation of dorsal midline tissues evaluated as the length of the *not* expression domain divided by the length of the whole-embryo, as previously described^36^.

### Embryo microsurgery (DMZ explants)

Following microinjection, early gastrula embryos (St. 10.5) were transferred to 1 X Modified Barth´s Saline (MBS) solution. Vitelline membranes were removed with forceps and DMZ explants were microsurgically isolated using hair tools^49,80^. To asses CE, the isolated explants were placed in plastic dishes, immobilized with a cover glass and cultured until co-cultured intact siblings reached late neurula stage (St. 19). The percentage of elongation was calculated as previously described^52,68^. For confocal microscopy, explants were prepared in Danilchik’s for Amy (DFA) culture media and mounted in custom acrylic chambers on glass prepared in advance with 20 μg/ml fibronectin (Sigma)^81^. The explants were imaged with a confocal microscope (SP5, Leica Microsystems). For Fry-GFP visualization in mesodermal cells, DMZ explants were allowed to adhere and heal for 30–40 min prior to imaging. For cell membrane visualization with mem-mScarlet, explants were cultured until sibling embryos reached late gastrula stage (St. 13/14). Cell orientation, polarity index, and cell area were calculated as previously described^49,66^ using image analysis software (ImageJ, https://fiji.sc/).

### Light-sheet fluorescence microscopy

Following microinjection, late blastula embryos (St. 9) were placed in pre-warmed (22 °C) 0.2% agarose (Biodynamics) prepared in 0.1 X MMR. Embryos were drawn into 4 cm long FEP tubes (Rotilabo, D-76185 inner diameter 1.58 mm, outer diameter 3.18 mm) with a needle and syringe^82^. The tube was plugged with solid 1% agarose and the samples were imaged using a custom-built light-sheet fluorescence microscope (LSFM)^39^. This microscope uses a cylindrical lens to generate a static Gaussian light-sheet that is focused into a sample using an illumination objective (Olympus UMPLFLN 10XW, 0.3 NA). The resulting light-sheet has a thickness of around 5 μm over a field of view of around 1 mm × 1 mm. The emitted fluorescence is collected using a detection objective (Olympus UMPLFLN 20XW, 0.5 NA) followed by a filter wheel, a tube lens and an sCMOS camera (Andor Zyla). Samples were scanned across the light-sheet using a stepper motor and the whole microscope was controlled using a custom-made graphical interface. 3D stacks of injected embryos were acquired upon visualization of the blastopore formation and every 3 min using an axial step of 5 μm over a distance of 400–600 μm. The resulting images were segmented and tracked using *ilastik*^40^*.* We analyzed the tracking results using custom-made Python scripts. We defined a cell’s instantaneous velocity $v$ as $v =\Delta r/\mathrm{\Delta t}$, where $\mathrm{\Delta r}$ is the cell’s spatial displacement over two consecutive frames and $\mathrm{\Delta t}$ the corresponding frame rate (3 min in all our experiments). We defined persistence of motion as the ratio between the linear distance traveled by a cell and the total length of its migration path^83^. In order to measure velocity and persistence of motion of dorsal superficial involuting marginal zone (IMZ) cells, we selected cells that: (i) were tracked for at least 45 min, (ii) moved a distance of at least 20 μm and (iii) were closer than 80 μm to the DBL at the last time point that they were tracked. The distance to the nearest neighbors was calculated as follows: for a single time-point (t = 150 min), the center of each nucleus as reported by *ilastik* was fed into the Delaunay algorithm (Python 3.6, SciPy 1.3) to obtain the list of nearest neighbors (NN). The Euclidean distance from each nucleus to their NN was calculated and then averaged. The distance of each cell from the blastopore lip was defined as the closest distance from the nucleus to a curve that was manually drawn over the lip for the frame of interest (region size = 30 μm; overlapping region size = 5 μm).

### BCR assays

Following microinjection, early gastrula embryos (St. 10.5) were transferred to 1 X Modified Barth´s Saline (MBS) solution. BCR assays to assess tissue separation behavior were performed as previously described^45^. Briefly, prechordal or deep layer ectodermal cells derived from uninjected or *fry*-MO injected embryos were dissected into small cell aggregates (test aggregates) and placed upon an uninjected BCR. Explants were immobilized with a cover glass and separation behavior was scored after 45 min.

### Statistical analysis

Numbers of embryos (n) and independent experimental replicates (N) for animal studies are stated in graphs. Statistical analysis for Figs. 1, 4c,d, 6c,d, 7 and Supplementary Fig. S7 were performed using *Chi*-square tests (**p < 0.001, ^†††^*p* < 0.001, ****,^††††^*p* < 0.0001). Statistical analysis for Figs. 4b,e, 5, 6e,f and Supplementary Fig. S6 and S2 was performed using two-tailed Mann Whitney *U*-tests (****p < 0.0001). Statistical analysis for Figs. 3, 4f, 8 and Supplementary Fig. S3 and S6 were performed using Kruskal–Wallis tests and means between groups were compared using Dunn's multiple comparisons tests (****p < 0.0001, **p < 0.01). For all statistical analyses we used Prism6, GraphPad Software, Inc.

## Supplementary Information

Supplementary Figure S1.

Supplementary Figure S2.

Supplementary Figure S3.

Supplementary Figure S4.

Supplementary Figure S5.

Supplementary Figure S6.

Supplementary Figure S7.

Supplementary Video 1.

Supplementary Video 2.

Supplementary Figure Legends.

Supplementary Table 1.
