# Supplementary figures and images for: Furry is required for cell movements during gastrulation and functionally interacts with NDR1
Source: Sci Rep. 2021 Mar 23;11:6607. doi: 10.1038/s41598-021-86153-x (PMC7987989; doi:10.1038/s41598-021-86153-x)

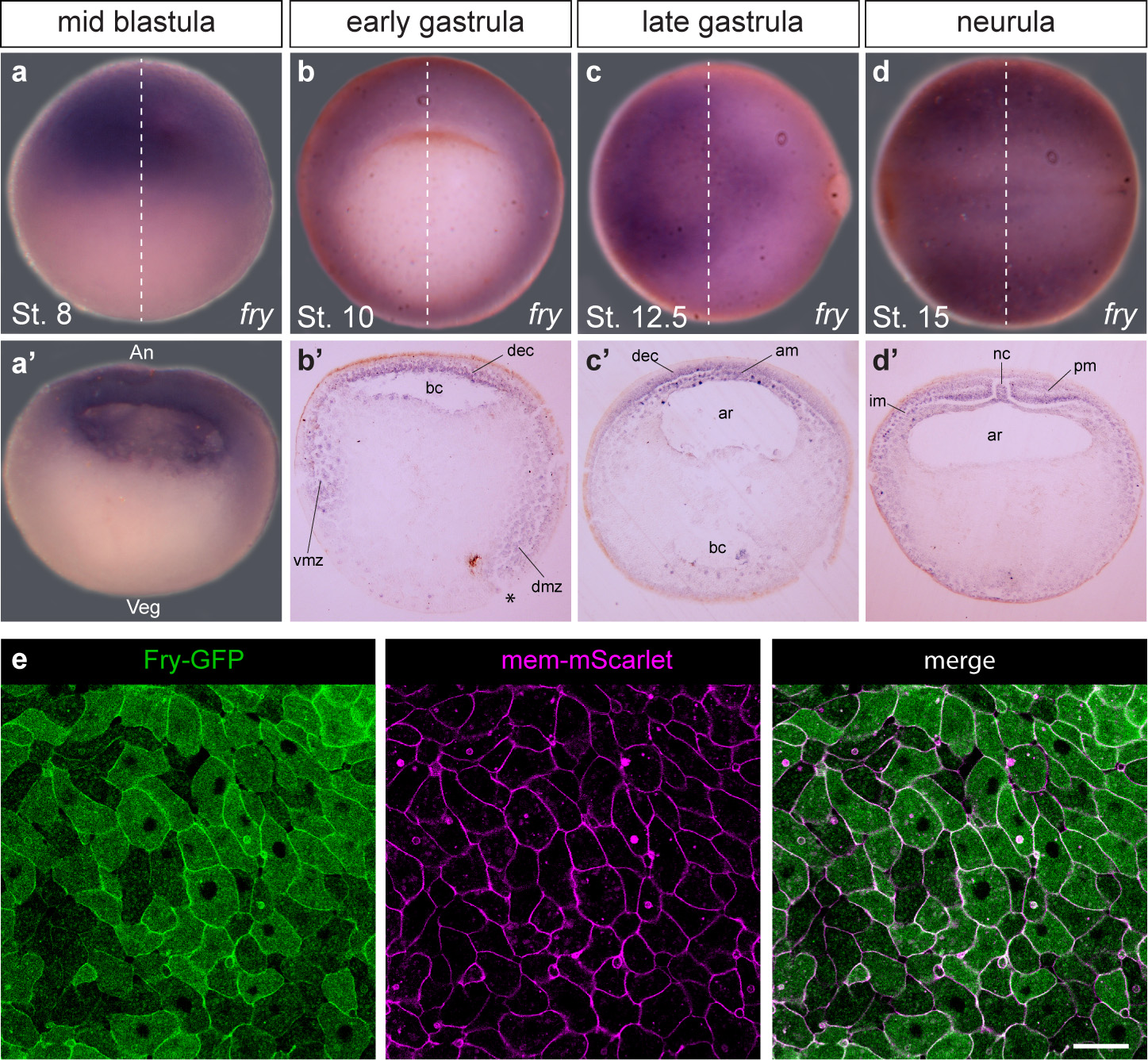

Supplement: Supplementary file 1 — Supplementary Figure S1. [file 41598_2021_86153_MOESM1_ESM.tif]

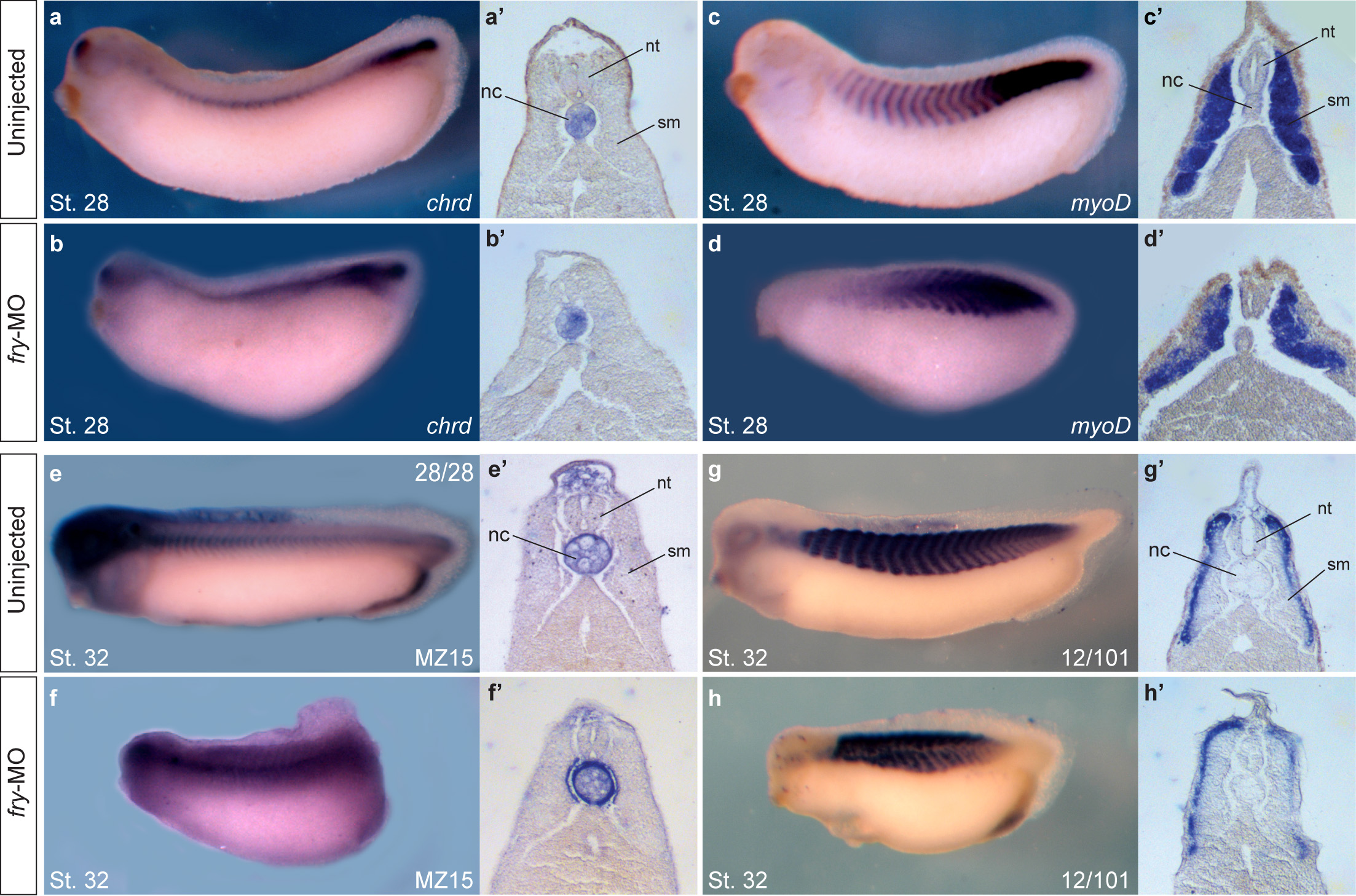

Supplement: Supplementary file 2 — Supplementary Figure S2. [file 41598_2021_86153_MOESM2_ESM.tif]

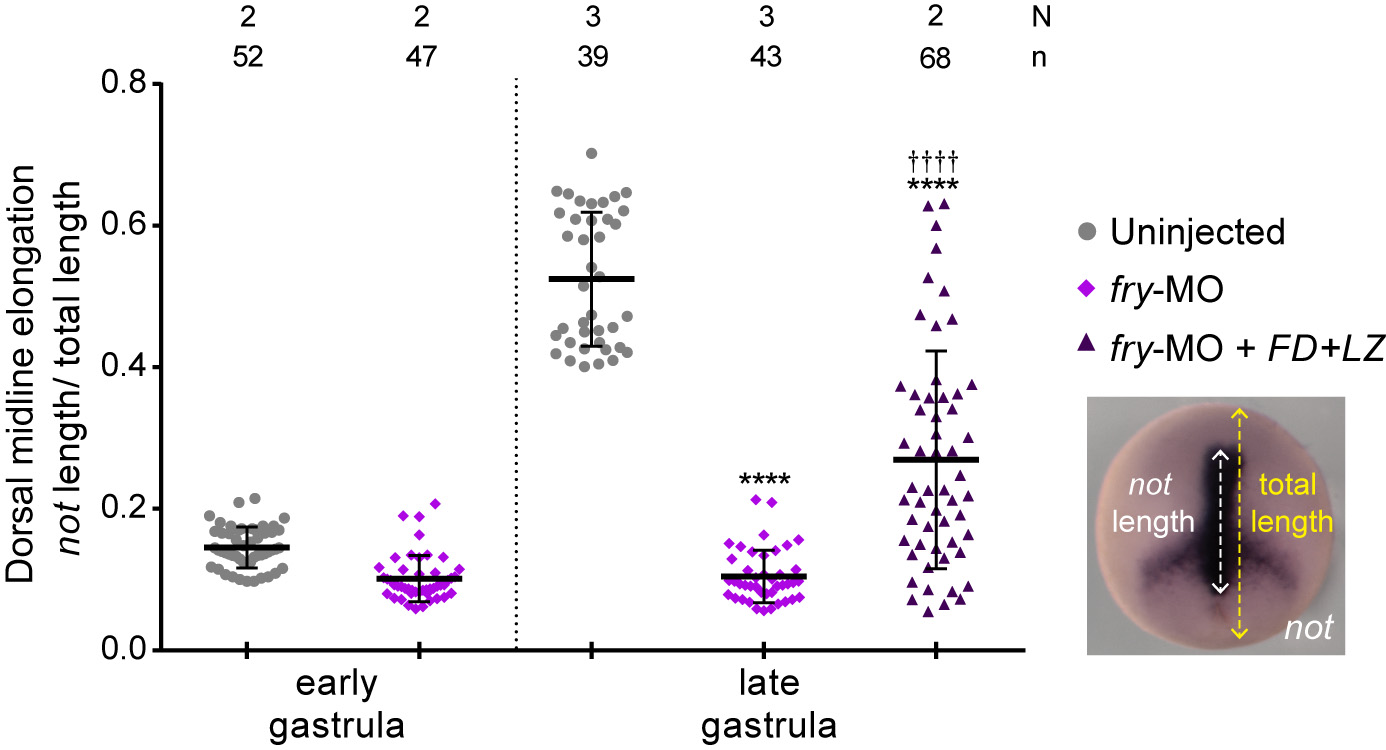

Supplement: Supplementary file 3 — Supplementary Figure S3. [file 41598_2021_86153_MOESM3_ESM.tif]

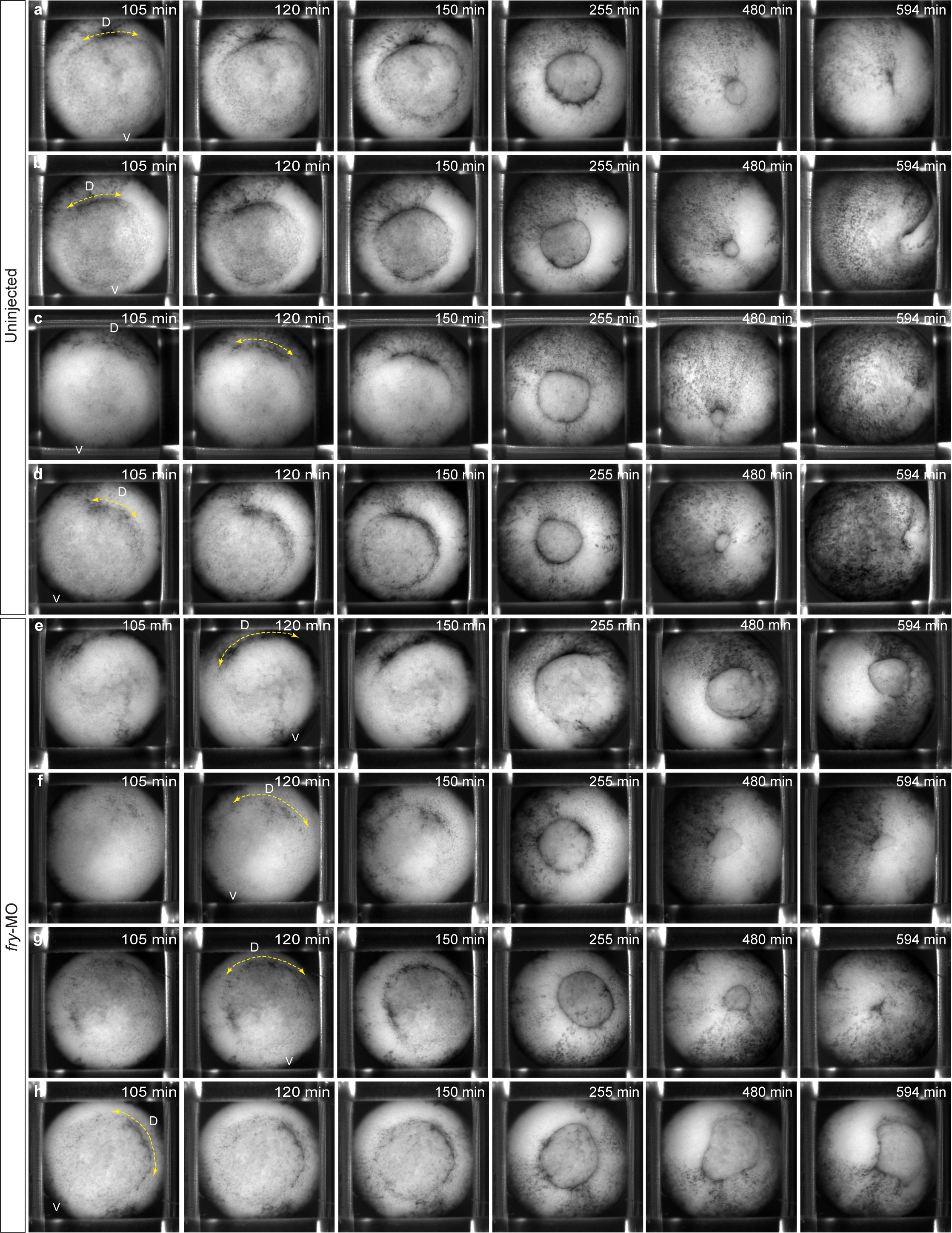

Supplement: Supplementary file 4 — Supplementary Figure S4. [file 41598_2021_86153_MOESM4_ESM.tif]

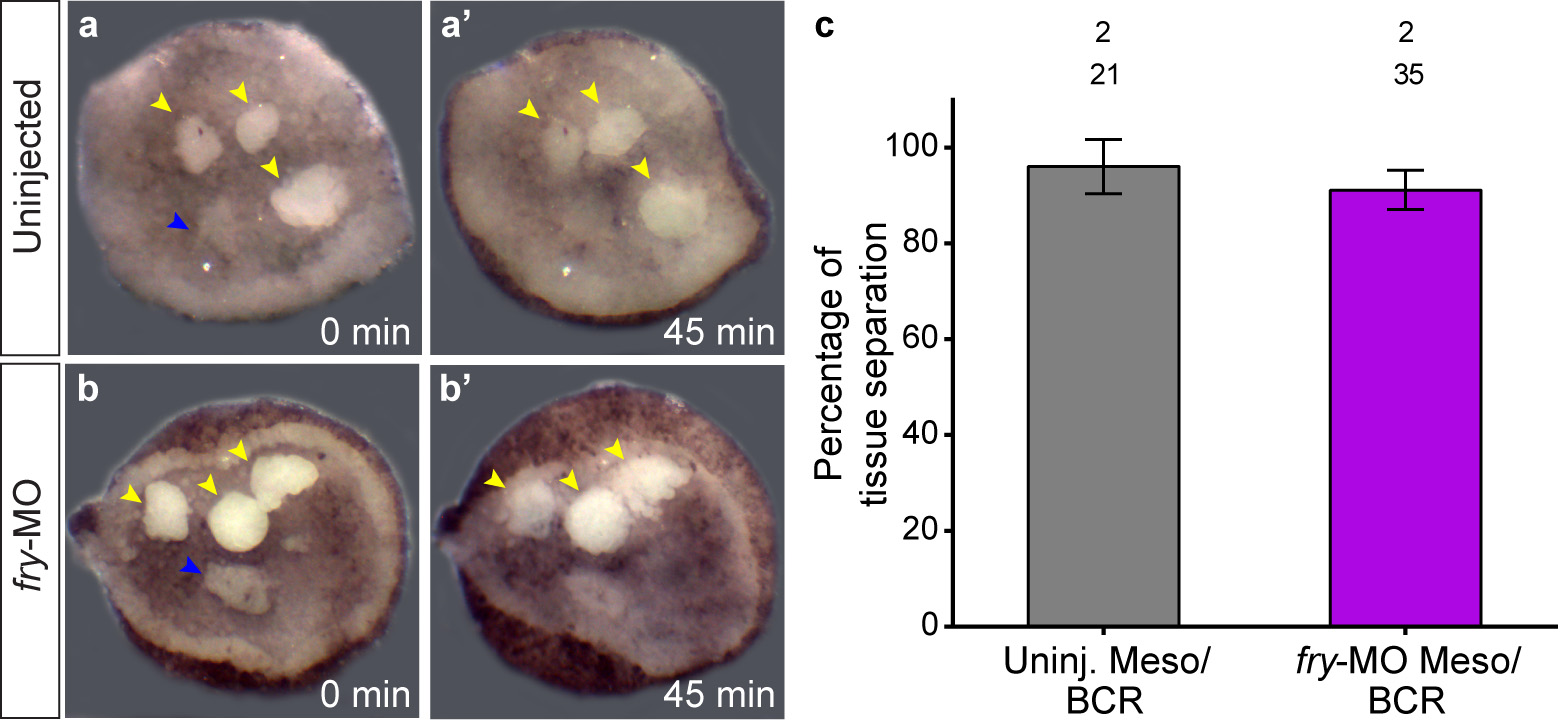

Supplement: Supplementary file 5 — Supplementary Figure S5. [file 41598_2021_86153_MOESM5_ESM.tif]

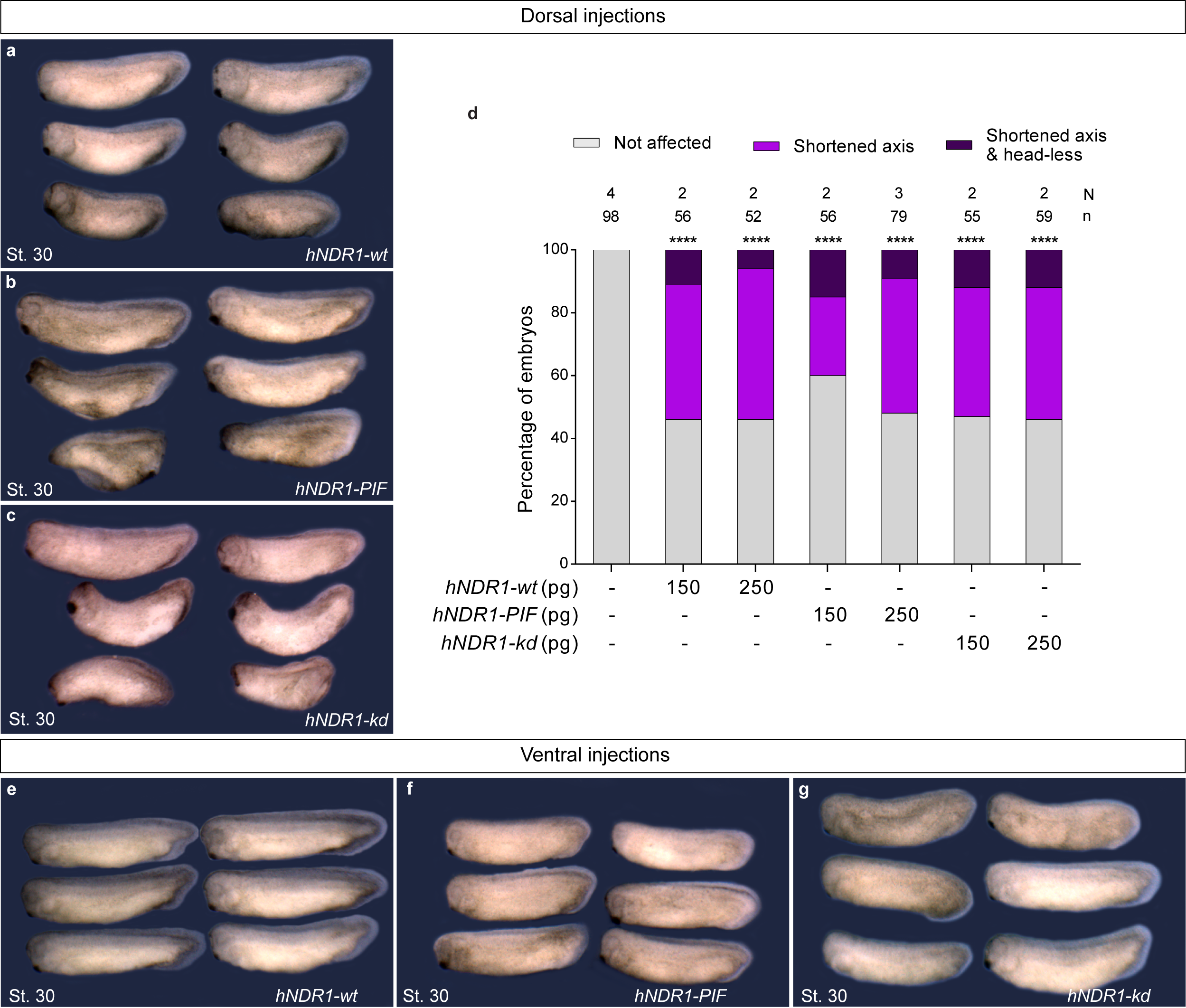

Supplement: Supplementary file 7 — Supplementary Figure S7. [file 41598_2021_86153_MOESM7_ESM.tif]
