# Supplementary material for: Furry is required for cell movements during gastrulation and functionally interacts with NDR1
Source: Sci Rep. 2021 Mar 23;11:6607. doi: 10.1038/s41598-021-86153-x (PMC7987989; doi:10.1038/s41598-021-86153-x)
Supplement: Supplementary file 10 — Supplementary Figure Legends. [file 41598_2021_86153_MOESM10_ESM.docx]

**Furry is required for cell movements during gastrulation and functionally interacts with NDR1**

Ailen S. Cervino^1^, Bruno Moretti^2^, Carsten Stuckenholz^3^, Hernán E. Grecco^2^, Lance A. Davidson^3,4,5^ and M. Cecilia Cirio^1,*^

**Supplementary Fig. S1. Fry expression and localization during gastrulation.** (**a**-**d**) *Fry* expression in *Xenopus* embryos. (**a**) Mid blastula stage embryo (St. 8). Dotted lines indicate the orientation of the hemisection (lateral view, animal pole up). (**a’**) Mid blastula stage embryo (St. 8) hemisection. Animal (An) and vegetal (Veg) poles are indicated. (**b**) Early gastrula stage embryo (St. 10.5) (vegetal view, dorsal up). (**c**) Late gastrula stage embryo (St. 12.5) (dorsal view). (**c**) Neurula stage embryo (St. 15) (dorsal view). Dotted lines indicate the orientation of the histological preparations showed in (**b’,c’,d’**). vmz: ventral marginal zone, dmz: dorsal marginal zone, bc: blastocoel, dec: deep layer of the ectoderm, am: axial mesoderm, ar: archenteron, nc: notochord, pm: paraxial mesoderm, im: intermediate mesoderm * indicates the position of the dorsal blastopore lip. Representative embryos are shown (**e**) Fry-GFP localization in dorsal mesoderm cells. Dorsal marginal zone explants were prepared from early gastrula embryos (St. 10.5) coinjected into both dorsal blastomeres of the 4-cell stage embryos with *fry-GFP* and *mem-mScarlet* mRNAs. Scale bar: 100 μm. A representative explant is shown (N = 2; n = 7) N: number of independent experiments, n: number of explants analyzed.

**Supplementary Fig. S2. Differentiated axial structures are present in dorsally *fry*-depleted embryos.** (**a,b**) Expression of *chrd* in stage 28 (St. 28) embryos by *in situ* hybridization. (**a**) Uninjected embryo (N = 2; n = 36). (**b**) *fry*-MO (15 ng) injected embryo (N = 2; n = 41). (**c,d**) Expression of *myoD* in the paraxial mesoderm of stage 28 (St. 28) embryos by *in situ* hybridization. (**c**) Uninjected embryo (N = 2; n = 40). (**d**) *fry*-MO (15 ng) injected embryo (N = 2; n = 43). (**e,f**) Notochord immunostaining with MZ15 antibody of stage 32 (St. 32) embryos. (**e**) Uninjected embryo (N = 2; n = 28). (**f**) *fry*-MO (15 ng) injected embryo (N = 2; n = 34). (**g,h**) Somitic muscle immunostaining with 12/101 antibody of stage 32 (St. 32) embryos. (**g**) Uninjected embryo (N = 2; n = 31). (**h**) *fry*-MO (15 ng) injected embryo (N = 2; n = 30). N: number of independent experiments, n: number of embryos. Embryos with representative expression or staining patterns are shown. All embryos analyzed showed positive staining. (**a’**-**h’**) Histological preparations (transverse sections) of embryos showed on a-h. nc: notochord; nt: neural tube; sm: somites. Note that both axial tissues are detected in *fry* morphant embryos, however they develop abnormally (e.g. reduced notochord diameter, discontinuous muscle staining).

**Supplementary Fig. S3. Chordamesoderm elongation requires Fry function.** Embryos at different stages were subject to *in situ* hybridization for *notochord homeobox*, *not.* Dorsal midline tissues elongation was measure on uninjected embryos, *fry*-MO (15 ng) injected embryos and *fry*-MO (15 ng) + *FD+LZ* mRNA (800 pg) coinjected embryos at early gastrula stage (St. 10.5) and late gastrula stage (St. 13). Right: Scheme of the metrics used: ratio of *not* expression domain length (*not* length, white) over whole-embryo length (yellow). N: number of independent experiments, n: number of embryos. The stage of injected embryos was established based on the stage of uninjected littermates. Data in the graphs is presented as means with standard deviation. Each point represents a single embryo. Statistical significance was evaluated using Kruskal-Wallis test and Dunn's multiple comparisons test (****,^††††^ *p*<0.0001). * represents the comparison to the uninjected group and † represents the comparison to the *fry*-MO injected group.

**Supplementary Movie S1. Blastopore closure in uninjected and *fry*-depleted embryos.** Time-lapse movies of gastrulating embryos. **(left)** Uninjected embryo (n = 12) (still frames **a** in Supplementary Fig. S5). **(middle** and **right)** *fry*-MO (15 ng) injected embryos (n = 12) (still frames **e** and **f** respectively in Supplementary Fig. S4). Note both embryos present blastopore closure delay relative to the uninjected embryo. Timestamp shows minutes elapsed. The movie begins at late blastula stage (St. 9) and continues through neurulation (St. 15). 1 frame every 3 minutes.

**Supplementary Fig. S4. Blastopore formation and closure in *fry*-depleted embryos.** Still frames from time-lapse movies (see Supplementary Movie S1) of gastrulating embryos at the indicated time-point (t) (vegetal view). Embryos were mounted at late blastula (St. 9) (t = 0). (**a-d**) Representative uninjected embryos (n = 12). (**e-h**) Representative *fry*-MO (15 ng) injected embryos (n = 12). Note that the blastopore formation is laterally expanded in *fry*-depleted representative embryos. Note all embryos present different degrees of blastopore closure delay. Only the embryo in **h** does not achieve blastopore closure. Dotted yellow arrows indicate the position and length of the dorsal blastopore lip when it is formed. D: dorsal; V: ventral.

**Supplementary Movie S2.** Time-lapse movies of representative embryos subjected to *light-sheet* fluorescence microscopy. (**left**) *H2B-eGFP* mRNA injected embryo (control). (**right**) *fry*-MO (15 ng) and *H2B-eGFP* mRNA coinjected embryo. 1 frame every 3 minutes.

**Supplementary Fig. S5. Fry depletion does not affect tissue separation behavior of dorsal mesoderm cells**. (**a**,**b**) BCR assay for separation behavior in *Xenopus* embryos. Dorsal mesoderm test aggregates (yellow arrowheads) or deep layer of the ectoderm test aggregates (blue arrowheads) were prepared from uninjected (**a**) or *fry*-MO (15 ng) injected embryos (**b**) and placed on an uninjected explanted BCR. Representative aggregates are shown. Tissue separation behavior was evaluated at time (t) = 45 min (**a’,b’**). Test aggregates that remained on explanted BCR surface are indicated by arrowheads. Note that ectodermal aggregates sunk on the surface of the explanted BCR while dorsal mesoderm test aggregates remain on the surface. (**c**) Quantification of BCR assay: percentage of mesoderm aggregates that exhibited tissue separation behavior. Data in the graph is presented as means with standard deviation. N: number of independent experiments, n: number of mesoderm test aggregates. Statistical significance was evaluated using two-tailed Mann Whitney *U*-test. No statistically significant differences were found between groups (p = 0.4258).

**Supplementary Fig. S6. *hNDR1-PIF* rescues the chordamesoderm elongation in *fry*-depleted embryos.** Neurula stage embryos (St. 15) were subject to *in situ* hybridization for *notochord homeobox*, *not.* Dorsal midline tissues elongation was measure as the length of *not* expression domain divided by the length of the whole-embryo of uninjected embryos, *fry*-MO (15 ng) injected embryos, *fry*-MO (15 ng) + *hNDR1-wt* mRNA (250 pg) coinjected embryos, *fry*-MO (15 ng) + *hNDR1-PIF* mRNA (250 pg) coinjected embryos, *fry*-MO (15 ng) + *hNDR1-kd* mRNA (250 pg) coinjected embryos. N: number of independent experiments, n: number of embryos. The stage of injected embryos was established based on the stage of uninjected littermates and confirmed by the presence of *not* expression in the anterior neuroectodermal domain. Data in the graphs is presented as means with standard deviation. Each point represents a single embryo. Statistical significance was evaluated using Kruskal-Wallis test and Dunn's multiple comparisons test (**** p<0.0001, ^††^ *p*< 0.01). * represents the comparison to the uninjected group and † represents the comparison to the *fry*-MO injected group.

**Supplementary Fig. S7. Dorsal overexpression of hNDR1 functional variants result in mild axis elongation defects.** (**a-d**) 4-cell stage *Xenopus* embryos were injected into both dorsal blastomeres as indicated and fixed at stage 30 (St. 30). (**a**) *hNDR1-wt* mRNA (250 pg) dorsally injected embryos. (**b**) *hNDR1-PIF* mRNA (250 pg) dorsally injected embryos. (**c**) *hNDR1-kd* mRNA (250 pg) dorsally injected embryos. Representative embryos are shown. (**d**) Quantitation of the percentage of dorsally injected embryos showing the different phenotypes: “Not affected”, “Shortened axis” or “Shortened axis & Head-less” phenotypes. Note that the axis phenotype is a mild axis elongation defect. Data on graph is presented as mean. N: number of independent experiments, n: number of embryos. Statistical significance was evaluated using *Chi*-square test. **** *p*<0.0001 indicates statistically significant differences relative to uninjected group. (**e-g**) 4-cell stage *Xenopus* embryos were injected into both ventral blastomeres as indicated and fixed at stage 30 (St. 30). (**e**) *hNDR1-wt* mRNA (250 pg) ventrally injected embryos (N = 2, n = 48). (**f**) *hNDR1-PIF* mRNA (250 pg) ventrally injected embryos (N = 2, n = 55). (**g**) *hNDR1-kd* mRNA (250 pg) ventrally injected embryos (N = 2, n = 36).
