# Supplementary material for: Furry is required for cell movements during gastrulation and functionally interacts with NDR1
Source: Sci Rep. 2021 Mar 23;11:6607. doi: 10.1038/s41598-021-86153-x (PMC7987989; doi:10.1038/s41598-021-86153-x)
Supplement: Supplementary file 11 — Supplementary Table 1. [file 41598_2021_86153_MOESM11_ESM.docx]

|  | Percentage of axis elongation | | |  |  |
| --- | --- | --- | --- | --- | --- |
|  | >80% | 80-50% | <50% | n | N |
| Uninjected | 100.0 | 0.0 | 0.0 | 51 | 2 |
| *fry*-MO | 0.0 | 51.7 | 48.3 | 60 | 2 |
| *fry*-MO + *hNDR1-wt* | 15.4 | 48.3 | 36.3 | 91 | 3 |
| *fry*-MO + *hNDR1-PIF* | 34.0 | 60.3 | 5.7 | 53 | 2 |
| *fry*-MO + *hNDR1-kd* | 1.9 | 51.9 | 46.2 | 52 | 2 |

**Supplementary Table S1.** **Analysis of axis elongation within three categories of shortened axis.** 4-cell stage *Xenopus* embryos were injected into both dorsal blastomeres as indicated and fixed at St. 30. The injected doses of *fry*-MO and *hNDR1* variants mRNAs were 15ng and 250pg, respectively. Embryos were scored according to their percentage of axis elongation relative to the mean of the uninjected group (considered 100% of axis elongation). Data on the table is presented as percentage. n: number of embryos; N: number of independent experiments.
